# Inhibition of IRES-dependent translation of caspase-2 by HuR confers chemotherapeutic drug resistance in colon carcinoma cells

**DOI:** 10.18632/oncotarget.24840

**Published:** 2018-04-06

**Authors:** Amel Badawi, Abhiruchi Biyanee, Usman Nasrullah, Sofia Winslow, Tobias Schmid, Josef Pfeilschifter, Wolfgang Eberhardt

**Affiliations:** ^1^ Pharmazentrum Frankfurt/ZAFES, Medical School, Goethe-University Frankfurt, Frankfurt/Main, Germany; ^2^ Institute of Biochemistry I, Goethe-University Frankfurt, Frankfurt/Main, Germany; ^3^ Present address: Institut für Biochemie, Westfälische Wilhelms-Universität Münster, Münster, Germany

**Keywords:** caspase-2, colon carcinoma cells, chemotherapeutic drug resistance, HuR, IRES translation

## Abstract

HuR plays an important role in tumor cell survival mainly through posttranscriptional upregulation of prominent anti-apoptotic genes. In addition, HuR can inhibit the translation of pro-apoptotic factors as we could previously report for caspase-2. Here, we investigated the mechanisms of caspase-2 suppression by HuR and its contribution to chemotherapeutic drug resistance of colon carcinoma cells. In accordance with the significant drug-induced increase in cytoplasmic HuR abundance, doxorubicin and paclitaxel increased the interaction of cytoplasmic HuR with the 5ʹuntranslated region (5ʹUTR) of caspase-2 as shown by RNA pull down assay. Experiments with bicistronic reporter genes furthermore indicate the presence of an internal ribosome entry site (IRES) within the caspase-2-5ʹUTR. Luciferase activity was suppressed either by chemotherapeutic drugs or ectopic expression of HuR. IRES-driven luciferase activity was significantly increased upon siRNA-mediated knockdown of HuR implicating an inhibitory effect of HuR on caspase-2 translation which is further reinforced by chemotherapeutic drugs. Comparison of RNA-binding affinities of recombinant HuR to two fragments of the caspase-2-5ʹUTR by EMSA revealed a critical HuR-binding site residing between nucleotides 111 and 241 of caspase-2-5ʹUTR. Mapping of critical RNA binding domains within HuR revealed that a fusion of RNA recognition motif 2 (RRM2) plus the hinge region confers a full caspase-2-5ʹUTR-binding. Functionally, knockdown of HuR significantly increased the sensitivity of colon cancer cells to drug-induced apoptosis. Importantly, the apoptosis sensitizing effects by HuR knockdown were rescued after silencing of caspase-2. The negative caspase-2 regulation by HuR offers a novel therapeutic target for sensitizing colon carcinoma cells to drug-induced apoptosis.

## INTRODUCTION

Evasion of apoptosis is a hallmark of tumor cells and a main cause for therapy resistance and relapse in cancer patients. Previous data implicate that spatiotemporal changes in the posttranscriptional regulation of gene expression by the action of micro RNAs and RNA-binding proteins (RBPs), represents an important mechanism how tumor cells cope with genotoxic stimuli [1]. Particularly, the ubiquitous turnover and translation regulatory protein human antigen R (HuR), a member of the embryonic lethal abnormal vision (ELAV) family, accounts as a key regulator of the posttranscriptional cell survival program relevant for chemotherapeutic drug resistance [2, 3]. HuR confers a broad anti-apoptotic program mainly through stabilization or translation of target mRNAs coding for prominent pro-survival factors including Bcl-2, prothymosin α, survivin, and the p53 inhibitor mouse double minute 2 (Mdm 2) [2]. In line with its broad cell survival activity, elevations in total and/or cytoplasmic HuR levels were found in a large variety of human cancers [4–7] and thereby can correlate with an increased therapy resistance [7]. Complementarily, HuR can repress translation of proteins as demonstrated for p27 [8], and for the human insulin growth factor-receptor (IGF-IR) [9]. In contrast to the stabilizing effects on target mRNAs which are mediated via HuR binding to the 3ʹuntranslated region (3′UTR), the constitutive inhibition of translation of the mentioned target genes is conveyed through an association of HuR with the 5ʹUTR of corresponding transcripts [8, 9]. HuR was shown to inhibit cap-dependent as well as internal ribosome entry site (IRES)-triggered translation which underlines the complex repertoire of posttranscriptional pathways by HuR [9, 10]. Previously, we reported the constitutive binding of HuR to the 5ʹUTR thereby suppressing caspase-2 translation in colon carcinoma cells [11]. Coincidentally, transient knockdown of HuR by siRNA can impair clonogenic colon carcinoma cell survival upon ionizing irradiation by an upregulation of caspase-2 translation [12]. The defined role of caspase-2 in apoptosis is still enigmatic since caspase-2 knockout mice did not show a clear phenotype [13]. In contrast, results from various studies implicate that caspase-2, by acting as an apical caspase, is critical for apoptosis-induced by several stimuli including DNA damage [14], reactive oxygen species (ROS) [15], mitotic catastrophe [16], and various chemotherapeutic drugs such as paclitaxel and doxorubicin [17, 18]. Similarily to the conventional initiator caspases (Caspases-8 and −9), pro-caspase-2 is activated by proximity-induced dimerization [19, 20]. In turn, ectopic expression of caspase-2 is sufficient to initiate autocatalytic activation of the enzyme [21]. In clear contrast to other initiator caspases, caspase-2 can not directly process effector caspases. Instead, caspase-2 activates the intrinsic apoptotic pathway upstream of mitochondria via cleavage of the pro-apoptotic protein Bid, a member of the Bcl 2 family resulting in the release of cytochrome c and second mitochondrial-derived apoptogenic proteins [22]. Despite that caspase-2 translation can be repressed by select microRNAs [23], reports about the mechanisms and pathophysiological relevance of caspase-2 translation are rare. In the present study, we elucidated whether the apoptosis-inducing potential of the two chemotherapeutic drugs doxorubicin and paclitaxel is potentiated upon HuR knockdown and whether the increase in sensitization may depend on caspase-2. Furthermore, we investigated the underlying mechanisms of the drug-induced modulation of the HuR-caspase-2 mRNA interaction. Our results indicate that colon carcinoma cells upon chemotherapeutic drug exposure induce HuR-dependent caspase-2 repression as a part of a so far unrecognized cell survival program by HuR.

## RESULTS

### HuR-silencing sensitizes colorectal carcinoma cells to drug-induced apoptosis

**Figure 1:**
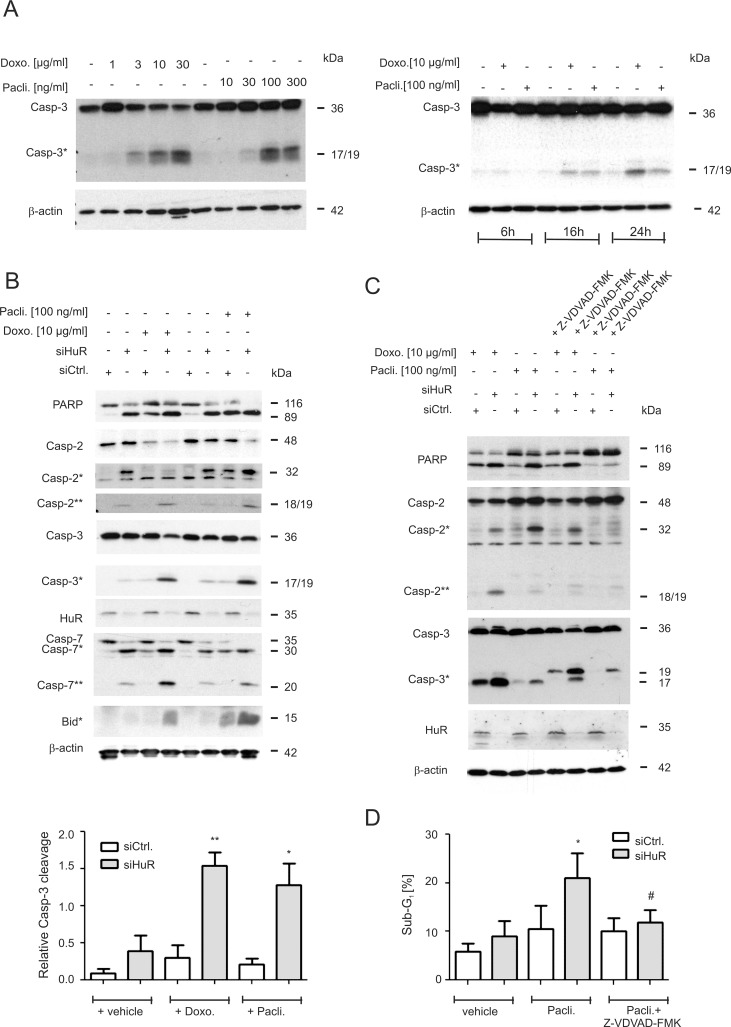
Increase in chemotherapeutic drug-induced apoptosis upon HuR knockdown in colon carcinoma cells (**A**) Concentration-(left panel) and time-dependent (right panel) activation of caspase-3 cleavage by chemotherapeutic drugs. DLD-1 cells were stimulated for the indicated time points with vehicle (−), or with the indicated doses of doxorubicin (Doxo.) or paclitaxel (Pacli.). Equal amounts of protein (20 μg) from total cell homogenates were subjected to SDS-PAGE and probed with anti-caspase-3 and anti-β-actin antibodies, respectively. The cleavage products of pro-caspase-3 (Casp-3) at 19 and 17 kDa are depicted by an asterisk (Casp-3*). Blots shown are representative for three independent experiments giving similar results. (**B**) Subconfluent DLD-1 cells were transfected with control siRNA duplexes (siCtrl.) or with siRNA duplexes targeting HuR (siHuR) for 48 h before cells were stimulated with either vehicle (−), or with the indicated doses of doxorubicin (Doxo.) or paclitaxel (Pacli.) and harvested 24 h later. Cleavage products of caspase-2, caspase-3, caspase-7, Bid (indicated by asterisks and double asterisks) and PARP, as well as knockdown efficiency of HuR was proven by Western blot analysis with β-actin was used as a loading control. A densitometrical analysis of cleaved caspase-3 levels in relationship to the content of mature caspase-3 is shown at the bottom of the figure. Data represent means ± SD (*n* = 3) ^*^P ≤ 0.05, ^**^P ≤ 0.01 siHuR vs. siCtrl. cells. (**C**) Subconfluent DLD-1 cells were transfected similar as described in panel (A) and subsequently stimulated with 10 μg/ml doxorubicin (Doxo.) or 100 ng/ml paclitaxel (Pacli.) either alone or in the presence of 50 μM of Z-VDVAD-FMK which was added 1 h prior to the administration of the chemotherapeutic drug. After 24 h, cells were lyzed for total cell extracts and the processing of PARP, caspase-2 and caspase-3 (cleavage products of caspases are indicated by asterisks and by double astersiks) and the knockdown efficiency of HuR subsequently monitored by Western blot analysis. Representative results of three independent experiments are shown. (**D**) Similarly, siRNA transfected cells were stimulated for 24 h with paclitaxel in the presence or absence of Z-VDVAD-FMK (50 μM) which was preincubated for 1 h before sub-G_1_ arrest was analyzed by flow cytometry (FACS) by propidium iodide (PI) staining. Values represent means ± SD (*n* = 3) and are depicted as percentage of cells in the sub-G_1_-phase ^*^P ≤ 0.05, siHuR vs. siCtrl. cells and ^#^*P* ≤ 0.05 Z-VDVAD-FMK plus paclitaxel treated siHuR transfectants vs. paclitaxel-treated siHuR transfectants and are depicted as percentage of cells in the sub-G_1_ phase.

Recently, we reported that silencing of the the RNA-binding protein HuR in colon carcinoma cells increases the sensitivity of colorectal carcinoma cells to ionizing radiation-induced cell death via the upregulation of caspase-2 [11]. In a first set of experiments, we tested whether this sensitizing approach would be also applicable to chemotherapeutic drugs. Therefore, we chose two clinically established anticancer compounds with different mechanisms of action including the topoisomerase-II inhibitor doxorubicin and the microtutuble stabilizing agent paclitaxel. Initially, we analyzed the induction of intrinsic apoptosis by monitoring caspase-3 cleavage in DLD-1 cells. Western blot analysis revealed a dose-and time dependent increase in caspase-3 cleavage in DLD-1 cells with both drugs (Figure 1A). Interestingly, when testing drug-induced effects additionally in RKO cells, we observed a higher sensitivity towards doxorubicin but lower sensitivity towards paclitaxel when compared with DLD-1 cells (Supplementary Figure 1A). For the following experiments, we chose medium doses of 10 μg/ml doxorubicin and 100 ng/ml paclitaxel and administered drugs usually for 24 h.

Next, the impact of HuR on drug-induced apoptosis was tested by employing transient HuR knockdown. We preferred to use a siRNA-mediated approach rather than a stable shRNA-mediated knockdown of HuR (shHuR)since the increase in caspase-2 protein levels upon inducible shHuR knockdown was only marginal and only transient (Supplementary Figure 1B). Previously, we reported that transient HuR knockdown caused a robust albeit transient increase in caspase-2 protein in DLD-1 cells mainly at 48 h of siRNA transfection although the knockdown efficacy of HuR remained stable [12]. An increase in caspase-2 protein upon HuR knockdown was also seen with siRNAs targeting another sequence of HuR (Supplementary Figure 1C) thus demonstrating that the induction of caspase-2 is not due to off-target effects. Furthermore, the HuR depletion-dependent increase in caspase-2 was only observed on the protein but not on mRNA levels (Supplementary Figure 1D). From these data it is tempting to speculate that colon carcinoma cells have evolved mechanisms which counterregulate an increase in caspase-2 levels either via an inhibition of caspase-2 translation and/or via an increased degradation of the enzyme. To achieve maximal sensitizing effects, the chemotherapeutic drugs were applied at a time point when caspase-2 levels peaked. For this reason, cells were routinely transfected for 48 h prior to the administration of the drugs. We found that concomitant with a moderate increase in full-length caspase-2, the levels of a caspase-2 cleavage product migrating at 32 kDa (Casp2*) was robustly increased upon HuR knockdown (Figure 1B). Importantly, in a clear contrast to the high drug sensitivity which we observed in untransfected cells (Figure 1A), caspase-3 cleavage in control siRNA transfectants was only weakly affected by both chemotherapeutic drugs but strongly induced upon siRNA mediated HuR knockdown (Figure 1B). Similarly, both drugs in combination with HuR silencing enhanced the appearance of a caspase-2 cleavage product (Casp-2**) at 18/19 kDa resulting from a second cleavage step in caspase-2 processing (Figure 1B) and a similar increase in drug-induced apoptosis upon HuR knockdown is indicated by the increased cleavage of poly ADP-ribose polymerase (PARP). An amplification of the intrinsic caspase cascade is further indicated by the increase in drug-induced caspase-7 and BH3 interacting domain death agonist (Bid) cleavage in HuR knockdown cells (Figure 1B).

### Role of caspase-2 in the HuR depletion-dependent apoptosis sensitization

In a next approach, we investigated the critical role of caspase-2 in HuR depletion-dependent sensitization of colon carcinoma cells to drug-induced apoptosis by employing Z-VDVAD-FMK, an inhibitor of caspases bearing a substrate which is preferentially cleaved by caspase-2 although these chemical inhibitors share similar cleavage motifs and therefore are not absolutely specific [24]. Preincubation of cells for 1 h with Z-VDVAD-FMK (50 μM) strongly reduced p17 levels but increased the content of the p19 caspase-3 cleavage product without causing a complete block of caspase-3 processing (Figure 1C). The effect was not due to siRNA transfection since the same changes in caspase-3 cleavage were observed in untransfected cells (Supplementary Figure 2A). In addition, Z-VDVAD-FMK impaired the generation of a doxorubicin-inducible caspase-2 (Casp2**) cleavage product at 18/19 kDa or, reduced the levels of a caspase-2 cleavage product at 32 kDa (Casp*) which accumulated upon HuR knockdown (Figure 1C, lane 4). These data implicate that depending on which chemotherapeutic drug is used for induction of apoptosis, both caspase-2 cleavage events are unequally impaired by Z-VDVAD-FMK. Similar effects on caspase-2 and −3 processing by Z-VDVAD were also observed in RKO cells (Supplementary Figure 2B). In addition, Z-VDVAD-FMK had a clear suppressive effect on drug-induced PARP cleavage as was most obvious after stimulation with paclitaxel (Figure 1C). Importantly, in accordance with the changes in caspases and PARP cleavage, the HuR depletion-dependent increase in paclitaxel treated cells accumulating in sub-G_1_ phase was significantly reduced by Z-VDVAD-FMK (Figure 1D). The latter finding underlines that caspase-2 activity is indispensable for apoptosis sensitizing effects by HuR knockdown in colon carcinoma cells.

**Figure 2:**
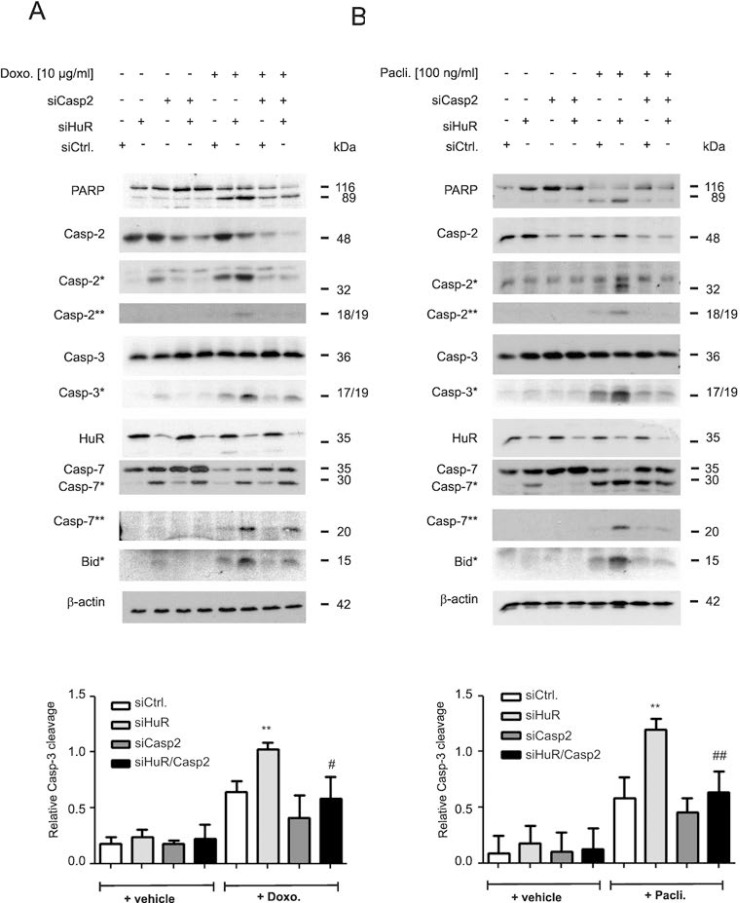
Caspase-2 is indispensable for chemotherapeutic drug-induced apoptosis in HuR depleted DLD-1 cells DLD-1 cells were transfected with control siRNA duplexes (siCtrl.) or with siRNA duplexes targeting caspase-2 (siCasp2) or HuR (siHuR) or both in combination for 48 h before stimulation with 10 μg/ml doxorubicin (**A**) or 100 ng/ml paclitaxel (**B**). After further 24 h, cells were harvested for total protein extraction and the cleavage of caspase-2, caspase-3, caspase-7, Bid (asterisks) and PARP as well as the knockdown efficiency of HuR and caspase-2 was determined by Western blot using β-actin as a loading control. The lower panels of the figure show densitometrical analysis of cleaved caspase-3 levels in relationship to the content of mature caspase-3 in the corresponding knockdown cell cultures. Data represent means ± SD (*n* = 3) ^**^P ≤ 0.01 siHuR vs. siCtrl. cells and ^#^*P* ≤ 0.05, ^##^*P* ≤ 0.01 siHuR/Casp2 vs. siHuR transfectants.

Owing to the lack of a caspase-2 specific inhibitor, the impact of caspase-2 in the HuR depletion-dependent apoptosis sensitization was additionally tested by siRNA-mediated silencing. Transfection of two different sets of caspase-2-specific siRNAs caused a similar reduction in caspase-2 levels (Supplementary Figure 2C). Intriguingly, the HuR depletion-mediated rise in drug-induced caspase-3 cleavage was significantly decreased by additional knockdown of caspase-2 independent of which chemotherapeutic drug was applied (Figure 2A and 2B). Likewise, the drug-induced cleavage of PARP, caspase-7 and Bid was impaired in HuR/caspase-2 double siRNA transfectants when compared to HuR knockdown cells (Figure 2A and 2B). A caspase-2-dependent increase in paclitaxel-induced caspase-3 cleavage upon siRNA-mediated knockdown of HuR was also found in HCT-15 cells (Supplementary Figure 2D) implicating that the sensitization of human colon carcinoma cells to apoptosis by HuR depletion is not a cell-type specific phenomenon.

**Figure 3:**
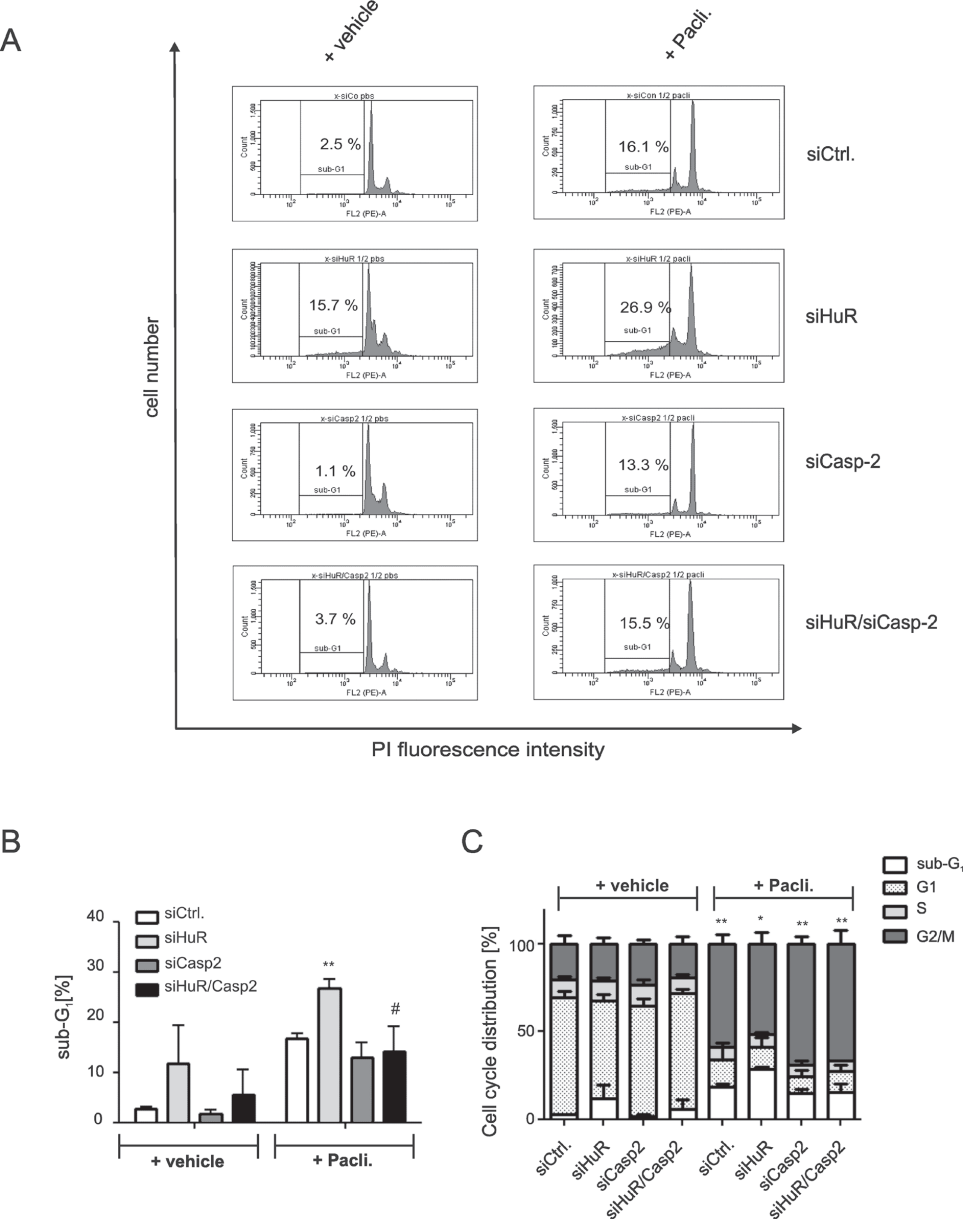
Knockdown of caspase-2 impairs HuR depletion-induced accumulation of DLD-1 cells in sub-G_1_ phase DLD-1 cells were transfected with control siRNA duplexes (siCtrl.) or with siRNA duplexes targeting caspase-2 (siCasp2) or HuR (siHuR) or alternatively, double transfected with HuR plus caspase-2-specific siRNA duplexes (siHuR/Casp2) for 48 h before cells were treated either with vehicle (+vehicle), or 100 ng/ml paclitaxel (+Pacli.). After further 24 h, cells were subjected to flow cytometric analysis for determination of sub-G_1_ arrest (**A**) Values in panel (**B**) represent means ± SD (*n* = 3) ^**^P ≤ 0.01 siHuR vs. siCtrl and ^#^*P* ≤ 0.05 siHuR/Casp2 vs. siHuR transfectants. The graph in (**C**) summarizes cell-cycle distributions from the same experiments. Values represent means ± SD (*n* = 3) ^*^P ≤ 0.05, ^**^P ≤ 0.01 paclitaxel-induced changes in G2/M.

**Figure 4:**
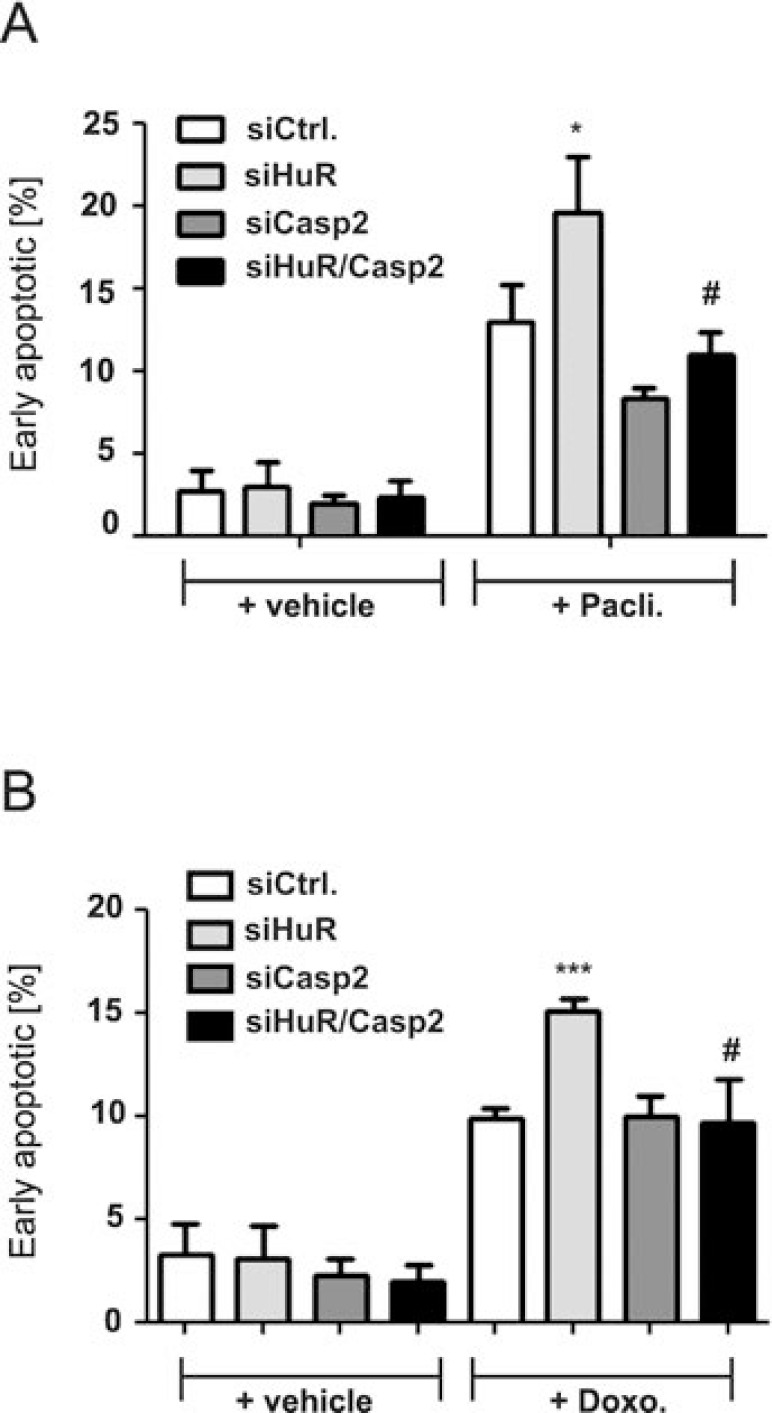
Knockdown of caspase-2 protects DLD-1 cells from HuR depletion-mediated sensitization for chemotherapy-induced early apoptosis DLD-1 cells were transfected with control siRNA duplexes (siCtrl.) or with siRNA duplexes targeting caspase-2 (siCasp2) or HuR (siHuR) or alternatively, double transfected with HuR plus caspase-2-specific siRNA duplexes (siHuR/Casp2) for 48 h before cells were treated either with vehicle (+vehicle), 100 ng/ml paclitaxel (**A**) or, 10 μg/ml doxorubicin (**B**). After further 24 h, cells were subjected to flow cytometic analysis for determination of early apoptosis by using annexin-V-FITC staining as described in the materials and methods section. Values represent means ± SD (*n* = 3) ^*^P ≤ 0.05, ^***^P ≤ 0.005 siHuR vs. siCtrl. and ^#^*P* ≤ 0.05 siHuR/Casp2 vs. siHuR transfectants.

A rescue from HuR depletion-dependent apoptosis by knockdown of caspase-2 was furthermore indicated by the reduced accumulation of HuR knockdown cells in the sub-G_1_ phase (Figure 3A, 3B). Monitoring for changes in cell cycle phase distribution by FACS furthermore revealed, that paclitaxel caused a significant increase in cells which accumulated in the G2/M phase (Figure 3C). To furthermore explore whether the HuR/Caspase-2-dependent changes in drug-induced apoptosis are already evident at the early phase of cell death, we employed fluorescently labelled Annexin V which is generally used as a probe to detect phosphatidylserine on the outer surface of the plasma membrane. Consistent with the results from sub-G_1_-phase analysis, both chemotherapeutic drugs clearly increased the basal early apoptosis (Figure 4A, 4B, Supplementary Figures 3 and 4). Similarily to changes in sub G_1_-phase, the knockdown of HuR caused a significant increase in drug-induced annexin V staining and importantly, the HuR depletion-dependent increase was totally impaired under caspase-2 compromised conditions independent of which chemotherapeutic drug was used (Figure 4A and 4B and Supplementary Figures 3 and 4).

**Figure 5:**
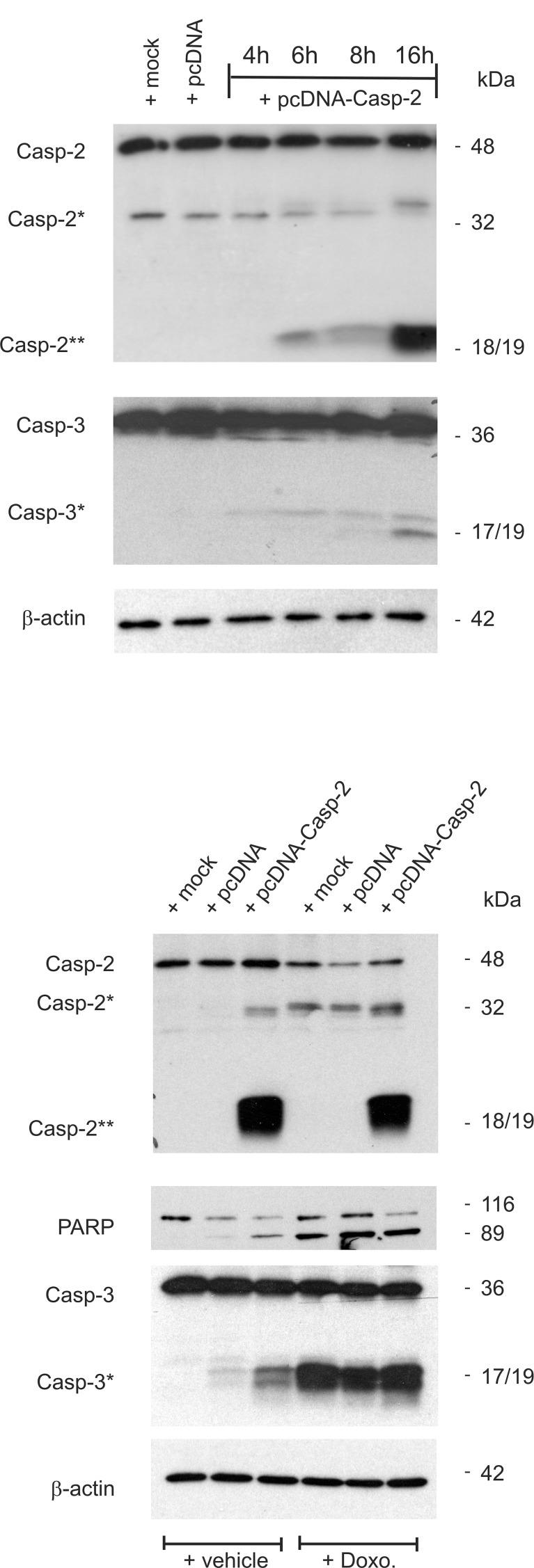
Overexpression of caspase-2 activates caspase-2 processing and is accompanied by an increased caspase-3 and PARP cleavage (**A**) Subconfluent DLD-1 cells were either mock transfected (mock), or transfected with 1 μg of empty pcDNA vector (pcDNA) for 24 h, or with the same amount of pcDNA3- Caspase-2 (pcDNA-Casp2) coding for full length caspase-2 for the indicated time period before cells were harvested for total cell extracts. 20 μg of total cell lysates were analyzed by Western blot for detection of unprocessed and cleaved caspase-2 and caspase-3 (asterisks). (**B**) Alternatively, cells were transfected as described in (A) before treated without (−Doxo.) or with 10 μg/ml doxorubicin (+Doxo.). Western blot analysis was used for detecton of unprocessed and cleaved caspase-2, caspase-3 (asterisks) and PARP, respectively. Representative results of three independent experiments are shown.

Conversely to the apoptosis inhibitory effects upon caspase-2 silencing, the overexpression of DLD-1 cells with pcDNA-caspase-2 caused a dose-dependent increase in basal caspase-2 and −3 cleavage (Figure 5A). Interestingly, the caspase-2 cleavage product at 18/19 kDa (Casp2**) was robustly induced by overexpression of caspase-2 without increasing the levels of the uncleaved pro-caspase (Figure 5A) indicating that overexpressed caspase-2 is completely processed. In contrast, treatment with doxorubicin caused a strong cleavage of caspase-3 but was unable to induce the generation of a caspase-2 cleavage product at 18/19 kDa (Casp2**). Conversely, ectopic expression of caspase-2 induced a moderate caspase-3 cleavage (Figure 5B) which indicates that caspase-2 does activate caspase-3 and not the reverse. Collectively, these results demonstrate that sensitiziation of colon cancer cells to chemotherapeutic drug-induced cell death by HuR knockdown critically depends on caspase-2 which further propagates the intrinsic apoptotic pathway induced by chemotherapeutic agents.

### Chemotherapeutic drug-induced increase in cytoplasmic HuR is concomitant with an increased HuR mRNA binding

**Figure 6:**
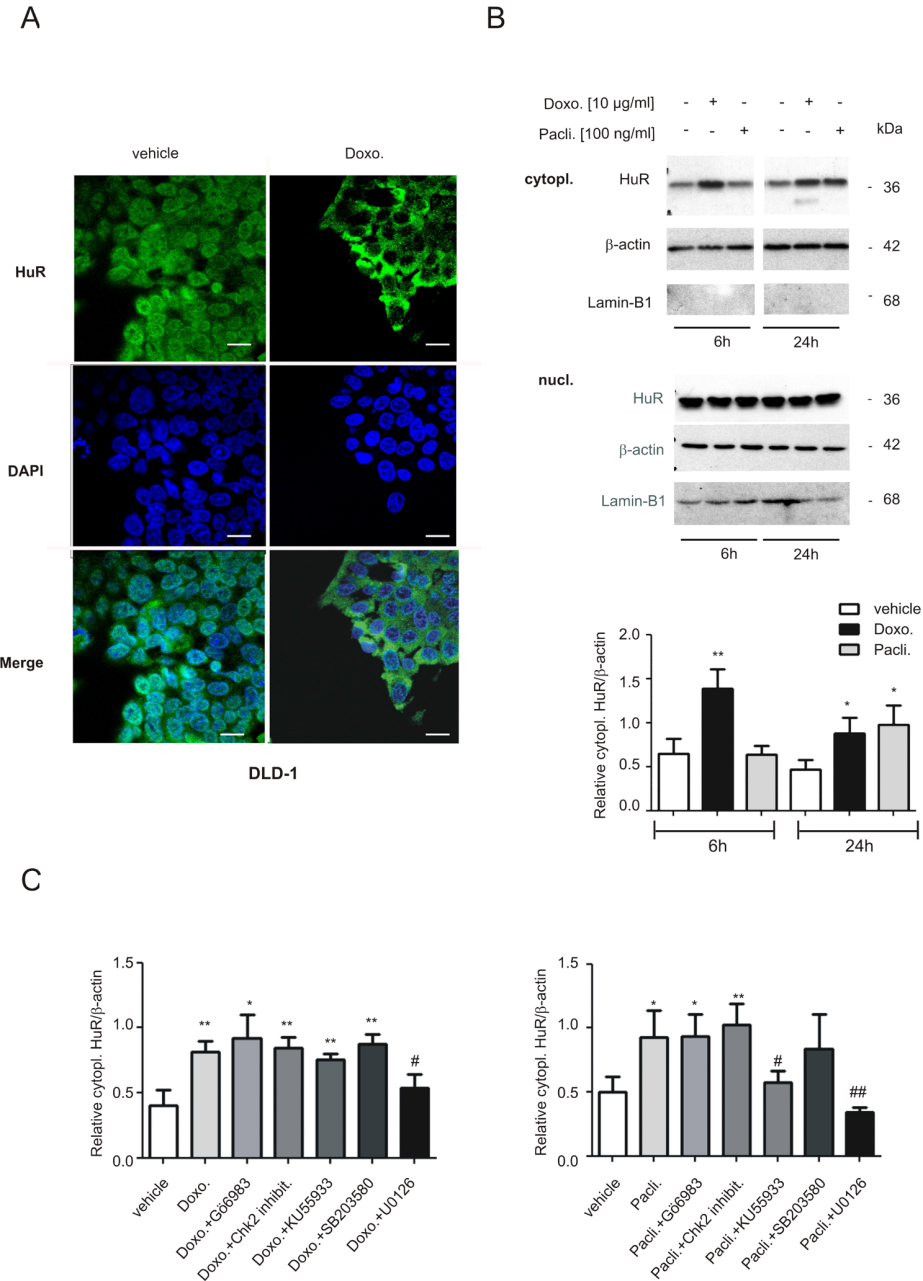
Modulation of nucleo-cytoplasmic HuR shuttling and binding to the 5ʹUTR-caspase-2 by chemotherapeutic drugs (**A**) Doxorubicin-induced nuclear export of HuR in human colon carcinoma cells by indirect immunofluorescence. Serum-starved DLD-1 cells were treated for 6 h with either vehicle, or with 10 μg/ml doxorubicin (Doxo.) before cells were fixed and successively stained with anti-HuR and anti-mouse Alexa-488 antibodies. Thereafter, DAPI was added to counterstain cell nuclei (blue panel). bar: 50 μm. (**B**) Time-dependent increase in cytoplasmic HuR content by chemotherapeutic drugs. Serum-starved DLD-1 cells were treated with doxorubicin (Doxo.) or paclitaxel (Pacli.) as indicated, before cells were lyzed for cytoplasmic cell lysates and cytoplasmic (cytopl.) or nuclear (nucl.) HuR levels were subsequently monitored by Western blot analysis. Loading of equal amounts of cytoplasmic extracts was ascertained by reprobing the blots with anti-β-actin antibody and Lamin-B1 was used as a nuclear marker protein. Values at the bottom represent means ± SD (*n* = 3) ^*^P ≤ 0.05, ^**^P ≤ 0.01 compared with the corresponding vehicles. (**C**) Cells were serum starved for 16 h before treated either 6 h with doxorubicin (left panel), or 24 h with paclitaxel (right panel) in the absence (vehicle) or presence of either Gö6983 (100 nM), the Chk2 inhibitor II (Chk2 inhibit. 2 μM), KU55933 (10 μM), SB203580 (10 μM) and U0126 (20 μM) as indicated. All inhibitors were preincubated for 30 min prior to the addition of the chemotherapeutic drug. Values in the graphs represent means ± SD (*n* = 3) ^*^P ≤ 0.05, ^**^P ≤ 0.01 compared with the corresponding vehicles and ^#^*P* ≤ 0.05, ^##^*P* ≤ 0.01 compared with doxorubicin (left panel) or paclitaxel (right panel)-induced conditions.

In the next set of experiments, we asked for the underlying mechanisms how both chemotherapeutic drugs could activate HuR triggered caspase-2 inhibition in colon carcinoma cells. First, we tested for drug-induced changes in nuclear HuR export which is thought as a major prerequisit for many mRNA regulatory HuR functions within the cytoplasm including the modulation of translation. By employing confocal-microscopy, we found that doxorubicin induced a strong relocalization of HuR from the nucleus to the cytoplasm in DLD-1 cells. The increase in export was most obvious at 6 h after drug administration (Figure 6A). Due to the disruption of the microtubule network by paclitaxel, which affected cell adherence to the coverslips, paclitaxel-induced effects could not be monitored by IF but were therefore analyzed by biochemical cell fractionation. In full accordance with results from fluorescence microscopy, doxorubicin caused a clear and persistant increase in cytoplasmic HuR levels (Figure 6B). Notably, the paclitaxel-induced increase in cytoplasmic HuR was stronger at 24 h (Figure 6B) indicating that both drugs may induce nucleo-cytoplasmic HuR shuttling by different signaling mechanisms. Again, similar effects were observed in RKO cells (Supplementary Figure 5A, 5B) implicating that drug-induced effects on HuR distribution are not cell-type specific. Next, we investigated the impact of different protein kinases, which are known to be involved in the regulation of HuR, by using different pharmacological inhibitors including Gö6983, a broad-spectrum protein kinase C (PKC) inhibitor, the checkpoint kinase 2 inhibitor II (Chk2 inhibit.), KU55933 an inhibitor of the ataxia-telangiectasia mutated (ATM) kinase, the p38 MAPK inhibitor SB203580 and U0126, an inhibitor of the MAPK kinase 1 and 2 (MEK1/2). Notably, only U0126 had a significant suppressive effect on drug-induced cytoplasmic HuR levels independent of which chemotherapeutic compound was used for HuR stimulation (Figure 6C). By contrast, KU55933 did only prevent paclitaxel-evoked cytoplasmic HuR abundance implicating that the ATM kinase is mainly involved in paclitaxel-induced HuR export. EMSA and supershift analysis furthermore revealed, that the increase in cytoplasmic HuR abundance is concomitant with an increase in RNA binding of drug-inducible complexes (C2, C3 and C4) to the 3ʹUTR of human cyclooxygenase-2 (COX-2) (Supplementary Figure 6), a prototypical HuR target in colon carcinoma cells [25]. These data indicate that the increase in cytoplasmic HuR by both chemotherapeutic drugs coincides with an increased HuR binding to AU/U-rich element (ARE) bearing mRNA.

**Figure 7:**
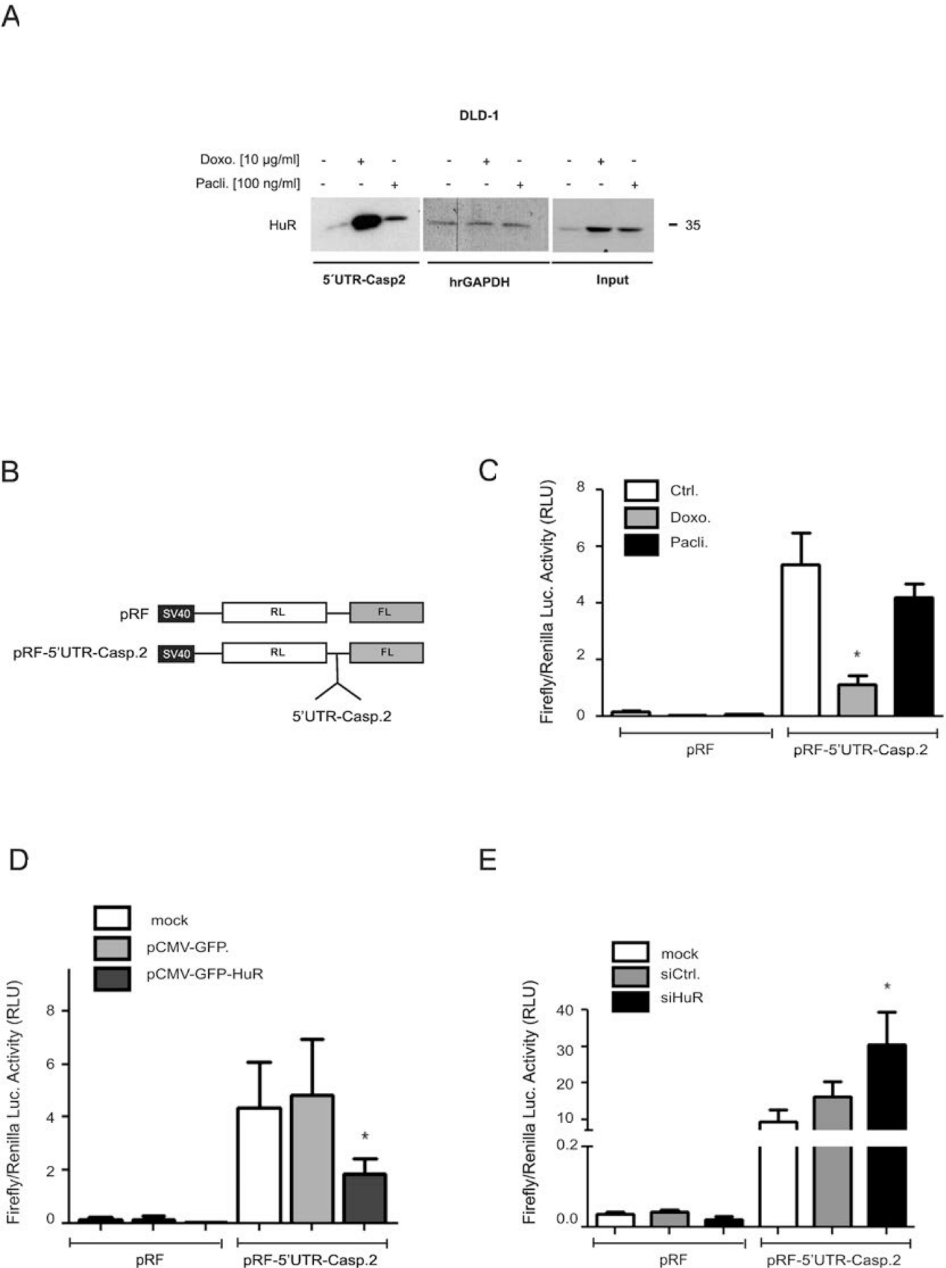
Drug-mediated increase in HuR binding to the 5ʹUTR-caspase-2 is concomitant with a modulation of 5ʹ-UTR-caspase-2 driven reporter gene activity A representative biotin pull-down assay demonstrating an increased HuR binding to the 5ʹUTR of caspase-2 by chemotherapeutic drugs is shown in (**A**). For pull-down assay, the biotinylated transcript encompassing 241 nucleotides of the 5ʹUTR of human caspase-2 was incubated with 300 μg of cytoplasmic cell extract from DLD-1 cells stimulated for 6 h with either vehicle (−), for 6 h with doxorubicin (Doxo.), or for 24 h with paclitaxel (Pacli.). Subsequently, HuR binding to the pull-down material was tested by Western blotting and the level of input protein was monitored by Western blot analysis (Input). The binding of the same amount of cell lysates to a biotinylated RNA (20 μg) from partial human reverse GAPDH (hrGAPDH) was assessed to confirm that changes in HuR binding are gene related. Representative results out of three independent experiments are shown. (**B**) Scheme of bicistronic plasmids used for reporter assays. *Renilla* (RL), *Firefly* (FL) (**C**). Subconfluent DLD-1 cells were transfected with 0.3 μg of either empty pRF or with the same amount of pRF bearing the full length 5ʹUTR of caspase-2 (pRF-5ʹUTR-Casp2) for 24 h before cells were treated with either vehicle (Ctrl.), with 10 μg/ml doxorubicin (Doxo.), or with 100 ng/ml paclitaxel (Pacli.). After 24 h, cells were lyzed and assayed for *renilla* and *firefly* activities. (**D**) Subconfluent DLD-1 cells were cotransfected as in (C) for 24 h either without DNA (mock) or, with 100 ng of empty pCMV-GFP vector or, alternatively with the same amount of pCMV-GFP-HuR coding for full length HuR. Subsequently, transfectants were lyzed and assayed for *renilla* and *firefly* activities. (**E**) DLD-1 cells were transfected without siRNA (mock), with control siRNA duplexes (siCtrl.) or, with siRNA duplexes of HuR (siHuR) for 48 h before additionally transfected with 300 ng of pRF or pRF-5ʹUTR-Casp2 for 24 h before cells were lyzed and assayed for *renilla* and *firefly* activites. Values for *firefly* luciferase in panels (B to E) were related to those for *renilla* luciferase and are depicted as relative light units (RLU). Data represent means ± SD (*n* = 4). ^*^P ≤ 0.05 compared with vehicle (C) or with mock transfected conditions (D, E).

To test whether in parallel, the drug-mediated changes in HuR shuttling and ARE-binding would also correlate with an increased HuR binding to the 5ʹUTR of caspase-2 bearing no AREs, we performed a streptavidin-tethered pull-down assay with *in-vitro* transcribed biotinylated mRNA encompassing the complete 5ʹUTR of caspase-2. Corresponding with the temporal changes in nucleo-cytoplasmic HuR distribution, doxorubicin caused a strong increase in cytoplasmic HuR binding to the 5ʹUTR of caspase-2 without affecting the constitutive binding to hrGAPDH (Figure 7A). In comparison, paclitaxel induced a weaker binding of HuR to the caspase-2-5ʹUTR. The fact that input levels from both samples were identical (Figure 7A) suggests that drug-induced effects on nucleo-cytoplasmic HuR shuttling do not fully correspond with changes in HuR´s RNA-binding affinity

### 5ʹUTR-driven reporter gene activity is negatively influenced by HuR

Based on our previous report indicating that the increase in caspase-2 protein upon HuR depletion is probably due to a modulation of cap-independent translation [11], we tested whether the increased HuR binding to 5ʹUTR-caspase-2 correlates with a decrease in IRES-dependent translation by utilizing a bicistronic reporter plasmid pRF-5ʹUTR-Casp2. Herein the 5ʹcistron (*renilla* luciferase) is expressed in a cap-dependent manner whereas expression of the 3ʹcistron (*firefly* luciferase) is under the control of the 5ʹUTR of caspase-2 and only translated in case that the 5ʹUTR bears a functional IRES (Figure 7B). Determination of the *firefly* to *renilla* luciferase ratio revealed a higher basal *firefly* activity in cells transiently transfected with pRF-5ʹUTR-Casp2 when compared to pRF transfectants indicating the presence of IRES elements within the 5ʹUTR of caspase-2 (Figure 7C). Treatment with both chemotherapeutic drugs reduced the relative *firefly* luciferase activity although paclitaxel had a weaker inhibitory effect on *firefly* luciferase activity (Figure 7C) which is in accordance with their different capacities to induce HuR-caspase-2 mRNA binding (Figure 7A). To test whether overexpression of HuR can act as a translation repressor through binding to the 5ʹUTR of caspase-2, a eukaryotic expression vector coding for wild-type HuR (pCMV-GFP-HuR) was cotransfected together with the bicistronic reporter plasmid (Figure 7D). Thereby, we found that expression of GFP-tagged HuR in contrast to the empty GFP vector caused a significant reduction in the relative *firefly* activity which further supports the notion that HuR acts as an inhibitor of caspase-2 translation. Conversely, RNAi-mediated HuR depletion caused a significant increase in relative *firefly* activity implicating that endogenous HuR suppresses caspase-2 translation via the 5ʹUTR (Figure 7E). To test whether chemotherapeutic drugs would affect the translation of caspase-2, DLD-1 cells were treated for 24 h with chemotherapeutic drugs before total cell lysates were layered on a sucrose gradient to sediment mRNAs according to their ribosome occupancy by ultracentrifugation. Since the ribosome distribution in doxorubicin-treated cells implicated a global inhibition in translation (Supplementary Figure 7A), we focused on paclitaxel mediated effects. Importantly, owing to the low yield of input material, single fractions from either subpolysomal ribonucleoproteins (RNP), light polysomes (LP) or heavy polysomes (HP) were pooled and subjected to semiquantitative RT-PCR (Supplementary Figure 7A). Paclitaxel induced a clear switch of caspase-2 mRNA from heavy polysomes to the subpolysomal RNP fractions without affecting the relative distribution of GAPDH mRNA thus implicating that caspase-2 translation is reduced by paclitaxel (Supplementary Figure 7A, lower panel). The fact that GAPDH mRNA showed an equal distribution between RNP and polysomal fractions points to an overall weak translational activity in DLD-1 cells which is also indicated by the flattened polysome profile shown in Supplementary Figure 7A. Furthermore, measurement of total mRNA levels by real-time PCR revealed that none of both chemotherapeutics caused significant changes in caspase-2 steady-state mRNA levels (Supplementary Figure 7B).

### Identification of HuR domains critical for 5ʹUTR binding

**Figure 8:**
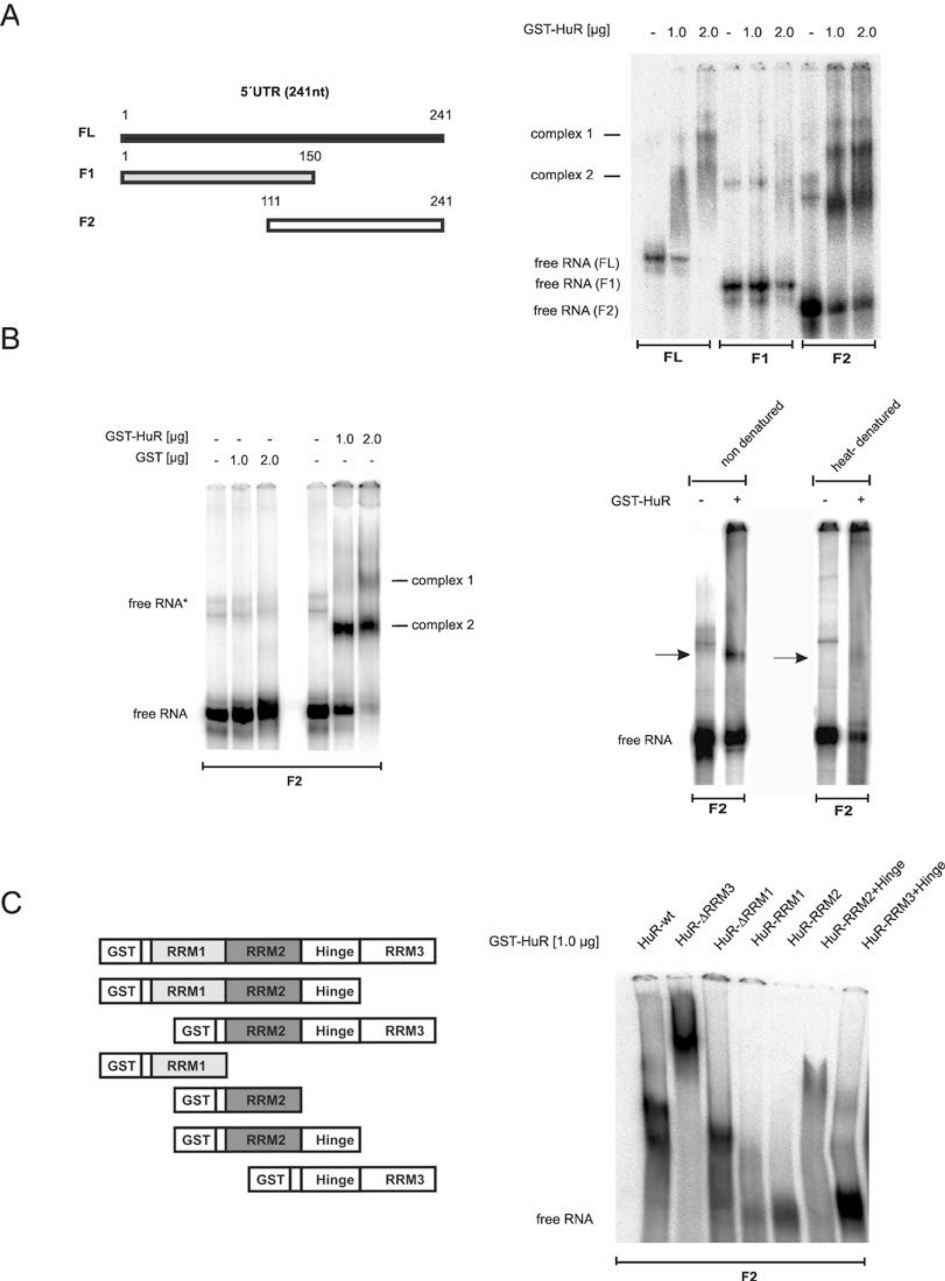
Characterization of *in vitro* binding of HuR to the 5ʹUTR of caspase-2 (**A**, *left panel*) Schematic representation of full-length and different fragments (F1, F2) encompassing the indicated regions of 5ʹUTR-caspase-2. Numbers show the position of nucleotides of the human caspase-2L mRNA (GenBankTM accession no.NM_032982). (**A**, *right panel*). *In vitro* binding of the indicated amount of recombinant GST-HuR to full-length or the indicated *in vitro* transcribed fragments of 5ʹUTR-caspase-2 (F1, F2) was assessed by EMSA using 6% native PAGE gels. The positions of unbound RNAs (free RNA) are indicated. Representative results of two independent experiments are shown. (**B**, *left panel*) *In vitro* binding affinity of GST. (**B**, *right panel*) *In vitro* binding of GST-HuR (1 μg) to *in vitro* transcribed F2 without a prior heat denaturation (non-denatured) or after 10 min of heat denaturation (heat-denatured) was assessed by EMSA on 6% native PAGE gels. The binding of complexes which disappeared after heat denaturation of the RNA is indicated by black arrows. Representative data of two independent experiments are shown. (**C**) Identification of HuR domains relevant for 5ʹUTR-caspase-2 binding. HuR deletion constructs coding for different GST-HuR fusion proteins are depicted on the left panel. A ^32^P-labeled *in vitro* transcribed RNA probe (F2) was incubated with 1.0 μg of the indicated fusion proteins and RNA-binding was monitored by EMSA. The data shown are representative for three independent experiments giving similar results.

To finally determine which HuR domain is critical for 5ʹUTR-caspase-2 binding, we compared the RNA binding affinity of recombinant HuR either to the full length 5ʹUTR (FL) or two shorter fragments (F1, F2) by EMSA (Figure 8A). Testing GST-tagged wildtype HuR revealed concentration-dependent appearance of two complexes (complex 1 and 2) to FL while no binding was observed with F1 encompassing nucleotides 1–150 of 5ʹUTR-caspase-2. In contrast, the strongest HuR binding was observed with the F2 probe which indicates that the critical HuR binding sites reside within a region from 111–241 (Figure 8A, right panel). In contrast, GST alone showed no RNA binding affinity (Figure 8B, left panel). The fact that HuR binding was lost after heat denaturation of the RNA probe furthermore implies that secondary structures within the RNA are critical for HuR binding (Figure 8B, right panel).

Next, we compared the binding affinities of full-length HuR and various HuR truncations (Figure 8C, right panel). The purity and migration of the different HuR proteins was confirmed by SDS-PAGE (Supplementary Figure 8A). Truncation of either the C-terminal RNA recognition motif (RRM) 3 or the N-terminal RRM1 had almost no effect on HuR binding capacity to the F2 fragment of caspase-2-5ʹUTR (Figure 8C, right panel). However, the lack of RRM3 (HuR-ΔRRM3) caused a strong shift in HuR migration probably due to an oligomerization of HuR. In a contrast to this results, previous reports have demonstrated that the RRM3 of HuR is a multifunctional domain which is critical for dimerization of the protein but in addition, a *bona fide* RNA interacting domain which preferably binds to U-rich stretches [26, 27]. Plausible explanations for these discrepant findings may arise from differences in the sequences and length of the RNAs used in different studies. Notably, in our experiments we have only analyzed the behavior of the ΔRRM3 HuR truncation but did not investigate the RNA binding affinity of the isolated HuR-RRM3 domain. By contrast, HuR-RRM1 similarly to HuR-RRM2 showed no binding to the F2 probe. Also, a fusion of the hinge region with RRM3 (HuR-RRM3+hinge) exerted only a weak RNA-binding affinity whereas a fusion of RRM2 plus hinge (HuR-RRM2+hinge) displayed a robust RNA binding to the 5ʹUTR of caspase-2 which is also reflected by the strong reduction of the free RNA probe (Figure 8C, right panel). These data indicate that RRM2 together with the hinge region is essentially involved in HuR binding to the caspase-2-5ʹUTR. Furthermore, the results suggest that the hinge region is presumably required for a correct folding of RRM2.

## DISCUSSION

In this study, we report on the sensitization of colon carcinoma cells to chemotherapeutic drug-induced apoptosis by transient HuR knockdown. The fact that the sensitizing effects by HuR depletion were observed in DLD-1, RKO and HCT-15 cells strongly implicates that the effects are not cell-type specific. Since HuR is known to promote cell survival by targeting various apoptosis regulatory factors [2] it was unexpected that apoptosis sensitizing effects by HuR knockdown were almost fully reverted by genetic or pharmacological depletion of only one HuR target mRNA. This finding underscores the strong impact of caspase-2 in drug-induced apoptosis in colon carcinoma cells.

Previously, we could show that HuR acts as a translational repressor of caspase-2 through its constitutive binding to the 5ʹUTR of caspase-2 without affecting the stability of the mRNA which is underscored by the fact that HuR did not constitutively bind to the 3ʹUTR of caspase-2 although it bears several prototypical HuR binding sites [11]. Here, we demonstrate a substantial increase in cytoplasmic HuR abundance in response to both chemotherapeutic drugs (Figure 6, Supplementary Figure 5) concomitant with an increased HuR binding to the 5ʹUTR of caspase-2 (Figure 7A) as well as to the 3ʹUTR of cox-2 (Supplementary Figure 6). This observation indicates that drug-induced HuR shuttling is accompanied by an increase in cytoplasmic HuR binding to subsets of mRNA targets which present common RNA-binding features for HuR independent of their relative position within the mRNA. Functionally, the increased HuR binding to caspase-2-5ʹUTR by doxorubicin and paclitaxel implies activation of a novel HuR-mediated survival mechanism. In the light of the well described genotoxic properties of both drugs, the HuR depletion-dependent increase in intrinsic apoptosis probably results from the impaired stimulus-induced apoptosis protection by HuR which is mainly due to the inhibition of caspase-2 translation. In accordance to this hypothesis, overexpression of caspase-2 induced the cleavage of caspase-3 and PARP even in the absence of doxorubicin (Figure 5). Conversely, activation of caspase-3 cleavage by doxorubicin was not accompanied by a complete caspase-2 cleavage which implicates that in colon carcinoma cells caspase-2 acts upstream of caspase-3. Activation of pro-caspase-2 after caspase-2 dimerization in many cases is achieved by the PIDDosome (*p*53-induced protein with a death domain) [28]. Interstingly, a previous study by Ando *et al.* could identify a novel PIDDosome-dependent caspase-2 activation platform in the nucleus which is induced by DNA damage [29]. Other modes of caspase-2 activation include the autocatalytic activation of caspase-2 by K^+^ efflux in response to pore-forming toxins [30], or by heat shock-triggered protein aggregation [31]. Previous data could demonstrate that activation of caspase-2 is critical for induction of apoptotic cell death by both doxorubicin [32] and by taxanes [33] implicating that caspase-2 may act as a sensor of drug-induced cell death signals and this sensor is increased upon HuR depletion. In contrast, activation of caspase-2 is impaired by the action of the intrinsic apoptose inhibitor 5 (API5/AAC11) which impedes the dimerization of the enzyme mainly through direct binding to the caspase recruitment domain (CARD) of caspase-2 [34]. The question whether HuR may additionally inhibit caspase-2 through increasing the expression of an intrinsic caspase inhibitor is an important issue which should be addressed by future experiments.

In line with reports describing HuR as a modulator of IRES-dependent translation, our data suggest that inhibition of caspase-2 is structurally due to a physical binding of HuR to its 5ʹUTR. Based on our previous observation that rapamycin, which specifically inhibits cap-dependent translation, did not affect caspase-2 expression in DLD-1 cells [11], we anticipated that HuR preferentially interferes with IRES-dependent caspase-2 translation. Importantly, apoptosis is known to cause a general inhibition of cap-dependent translation [35]. However, a group of mRNAs with functional IRES elements in their 5ʹUTRs is refractory to translational repression among them mRNAs coding for prominent apoptosis regulatory factors such as XIAP [36], Apaf-1 [37] and c-myc [38]. A negative interference of HuR with IRES-dependent translation of caspase-2 is indicated by the following observations: 1) Using a bicistronic vector, we demonstrate that overexpression of HuR, or stimulation with chemotherapeutic drugs strongly reduced the high constitutive 5ʹUTR- caspase-2-driven *firefly* luciferase activity. Although the 5ʹUTR in the pRF vector may principally act as 3ʹUTR for *renilla* luciferase mRNA but this scenario is quite unlikely since the sequence of caspase-2-5ʹUTR contains no AREs. 2) Endogenous as well as recombinant HuR exerted a robust binding affinity to the 5ʹUTR of caspase-2 (Figure 7A, Figure 8) which can form energetically stable secondary structures with various hairpins as indicated by using *mfold* software (Supplementary Figure 8B). Similarily to the full length 5ʹUTR, *mfold* analysis indicates that the F2 fragment, which displayed the highest HuR binding affinity in EMSA (Figure 8A), bears several putative hairpin structures which may act as HuR binding sites. Considering the proposed length of a minimal IRES which is in the range of 100–1000 nucleotides [35], a mapping of the IRES within the caspase-2-5ʹUTR by deletion mutation was not followed up further. 3) Denaturation of the RNA strongly impaired HuR binding to the caspase-2-5ʹUTR (Figure 8B, right panel). In accordance to our data, a negative effect on IRES-dependent translation by HuR has so far been demonstrated for IGF-IR [9] and p27^kip1^ [8]. Mechanistically, HuR is known to compete with an IRES *trans*-acting factor (ITAF) for a common IRES binding [9]. Alternatively, HuR itself may act as a negative ITAF that may hinder the recruitment of the 40S ribosome by inducing a conformational switch in the RNA [9]. Further studies are needed to address this important issue.

Taken together, our findings indicate that chemotherapeutic drugs independent of their specific antitumor activity through activating HuR export and cytoplasmic caspase-2-5ʹUTR binding suppress IRES-dependent translation of caspase-2 presumably as part of a posttranscriptional survival program utilized by colon carcinoma cells.

## MATERIALS AND METHODS

### Reagents and antibodies

All cell culture media, supplements and modifying enzymes were purchased from Invitrogen (Karlsruhe, Germany) if not otherwise indicated. Doxorubicin was from Biotrend Chemicals (Cologne, Germany), paclitaxel, and propidium iodide were obtained from Sigma-Aldrich (Taufkirchen, Germany). Gö6983, KU55933, SB203580, U0126 and Chk2 inhibitor II were from Merck (Darmstadt, Germany). The caspase inhibitors Z-VDVAD-FMK and Z-VAD-FMK were derived from R & D Systems (Wiesbaden, Germany). The following antibodies were used in this study: anti-HuR (sc-5261) and anti-Lamin B1 (sc-20682) from Santa Cruz (Heidelberg, Germany), anti-caspase-2 (#611022, BD Biosciences, Heidelberg, Germany), anti-caspase-3 (#9662), anti-caspase-7 (#9494) anti-PARP (#9542) and anti-Bid (#2002) antibodies were from Cell Signaling (Frankfurt, Germany), and anti-β-actin (#A2228) from Sigma-Aldrich. The goat anti-rabbit (sc-2054) and goat anti-mouse (sc-20559) HRP-linked antibodies were from Santa Cruz and Alexa Fluor 488 goat anti-mouse was from Life Technologies (Darmstadt, Germany). Radionucleotides were from Perkin Elmer (Rodgau, Germany) and the ECL system and Hyperfilms were from GE Healthcare (Freiburg, Germany).

### Cell lines and cell culture

The human colorectal carcinoma cell lines DLD-1 and HCT-15 were obtained from the German Collection of Microorganisms and Cell Cultures (Braunschweig, Germany) and the p53 wild-type cell line RKO was from the American Type Culture Collection (LGC-Promochem, Wiesbaden, Germany). Cells were grown in Dulbecco's modified Eagle's medium or in RPMI supplemented with 10% heat-inactivated fetal calf serum, 100 U/ml penicillin, and 100 μg/ml streptomycin.

### RNA interference

Transfection of subconfluent cells with siRNAs was performed as described previously [12]. To minimize off-target effects, we used a mixture of different siRNAs complementary to distinct regions of the mRNA. Briefly, 50 nM of a mixture of FlexiTube siRNAs for human HuR (SI00300139, SI03166436, SI103246551 and SI03246887, “siHuR#1”) or small interfering RNA (siRNA)-duplexes from Santa Cruz siRNA-HuR, sc-35619 (“siHuR#2”), or the same amount of FlexiTube siRNA for caspase-2 (SI00299551, “siCasp2#1”) from Qiagen (Hilden, Germany) or, alternatively, a siRNA-caspase-2, sc-29236 from Santa-Cruz (“siCasp2#2”) were used for single knockdown of corresponding genes. For silencing of both genes, both siRNAs (siHuR#1 plus siCasp2#1) were givend in combination each at a concentration of 25 nM. 48 h after transfection, the cells were treated for specific applications before lyzed for Western blot anaylsis.

### Stable HuR knockdown

Stable knockdown of HuR was achieved by using the ELAVL1-specific human TRIPZ™ lentivirus inducible shRNA (V3THS_331821, V3THS_331823, V3THS_331824) from Dharmacon. As control, cells were transduced with lentiviral particles bearing a non-targeting control shRNA (RHS_4750). Transfection of DLD-1 cells was performed by using the DharmaFect kb DNA transfection reagent (Dharmacon) by following the manufacturer´s instruction. For selection of virus-transduced cells, the cells were grown in selective medium containing puromycin (1 μg/ml). The time course of transgene expression was monitored (24, 48 and 72 h) by Western blot analysis and surviving cell clones were propagated for further applications.

### Quantitative RT-PCR

Total RNA was extracted from cells by using the Tri reagent from Sigma-Aldrich. First strand cDNA synthesis and two-step PCR were performed using a Taqman (ABI 7500) from Perkin Elmer (Rodgau, Germany) as described previously [11].

### Western blot analysis

Whole cell lysates were prepared as described previously [11]. Briefly, cells were lyzed in ice cold lysis buffer (137 mM NaCl, 20 mM Tris-HCl pH 8.0, 5 mM EDTA pH 8.0, 10% glycerol and 1% Triton X-100) and protease inhibitor mix (Roche, Mannheim, Germany) and subsequently subjected to several freeze-thaw cycles by using liquid nitrogen. 30 μg of total cell lysates were prepared in SDS sample buffer and resolved by 12%-15% SDS-PAGE before proteins were transferred onto nitrocellulose membranes. Detection of proteins was done by using specific antibodies and the appropriate secondary antibodies before signals were visualized by the ECL system.

### Plasmid construction

The bicistronic vector pRF-5ʹUTR-Casp2 bearing the complete 5ʹUTR (241 nucleotides) of caspase-2 was generated by amplification of a corresponding cDNA fragment from the plasmid pCR2.1-5ʹUTR-caspase-2 [11] by using *SpeI* and *NcoI* restriction sites as followed: *NcoI*-flanked (underlined) forward (fwd.) primer 5ʹ-ATA TCCATGGCT TTT GTC TGT CCG CCG AGC A-3ʹ and *SpeI*-flanked (underlined) reverse (rev.) primer 5ʹ-ATA TACTAGTTT CCC GCT TTT CCC GGG CTC T-3ʹ, corresponding to a region from nucleotides 1-241 of the human caspase-2L mRNA (GenBankTM accession no. NM_032982) directly into *SpeI*/*NcoI* cut bicistronic vector pRF (kindly provided by Prof. A. Willis) [37, 39]. For EMSAs, the F1 fragment was cloned by ligating a shorter fragment from nucleotides 1–150 with the *HindIII*-flanked (underlined) fwd. primer 5ʹ-ATA TAA GCT TCT TTT GTC TGT CCG CCG AGC A-3ʹ and *NcoI*-flanked (underlined) rev. primer 5ʹ-ATA TCC ATG GCG GAC GCA CAC TGC GCC TGC G-3ʹ, corresponding to a region from nucleotides 1–150 of the human caspase-2L mRNA into *HindIII/NcoI* cut pCR2.1-5ʹUTR-caspase-2 vector. Accordingly, the F2 fragment which was also used for EMSA was cloned by ligation of a 130 bp fragment with the *HindIII*-flanked (underlined) fwd. primer 5ʹ-ATA TAA GCT TGG GCG CAG GCG CAG GCG CAG T-3ʹ and *NcoI*-flanked (underlined) rev. primer 5ʹ-ATA TCC ATG GTT CCC GCT TTT CCC GGG CTC T-3ʹ, corresponding to a region from nucleotides 111–241 of the human caspase-2L mRNA into *HindIII/NcoI* cut pCR2.1-5ʹUTR-caspase-2 vector. For *in vitro* transcription, the same fragments were cloned into pCR2.1 plasmid downstream from the T7 promoter and linearized with *BamHI*.

Expression plasmids for purification of GST-tagged wild-type HuR and HuR truncations were a kind gift from Prof. Dr. Chemnitz (University of Göttingen, Germany) and were cloned as described previously [40].

### Polysomal fractionation

Isolation of RNA from different subpolysomal and polysomal fractions was performed as described previously [41]. Briefly, 5–7 × 10^6^ cells were cultured on 15 cm dishes one day prior to stimulation and followed by polysomal fractionation. 15 min prior to cell harvesting, the cells were treated with cycloheximide (CHX, 100 μg/ml) and lyzed in polysome buffer (140 mM KCl, 20 mM Tris-HCl pH 8.0, 5 mM MgCl_2_, 0.5% NP40, 0.5 mg/ml heparin, 1 mM DTT, 100 U/ml RNasin [Promega], 100 μg/ml CHX). After lysis, the cells were loaded onto 10%–50% continuous sucrose gradients which were centrifuged at 35,000 rpm for 2 h at 4° C. Gradients were subsequently collected in 1 ml fractions by using a gradientStation (Biocomp) and absorbance was measured at 254 nm. RNA was isolated by sodium acetate and isopropanol precipitation and further purified by using the Nucleo Spin RNA Kit (Machery-Nagel) following the manufacturer´s instructions. RNA was quantified using a NanoDrop Spectrophotometer (Thermo Fisher Scientific). After cDNA synthesis, individual mRNAs contents were measured by semiquantitative RT-PCR using GoTaq hot start polymerase (Promega). Primer sequences used for PCR were as followed: Caspase-2 fwd.: 5ʹ-ACA GGG GAC GCA GGA TAT TGG GA-3ʹ, Caspase-2 rev.: 5ʹ-GGT GGC CTT GCT TGG TCT CCC T-3ʹ; GAPDH fwd.: 5ʹ-TGC ACC ACC AAC TGC TTA GC, GAPDH rev.: 5ʹ-GGC ATG GAC TGT GGT CAT GAG-3ʹ. The PCR products were separated on a 1% agarose gel containing GelRed (Biotium).

### Reporter gene assays

DLD-1 cells were transiently transfected in 60 mm dishes with 0.3 μg of bicistronic vectors (pRF, pRF-5ʹUTR-Casp2) by using the Effectene transfection reagent (Qiagen, Hilden, Germany). 48 h later, cells were stimulated with chemotherapeutic drugs for further 6 h (doxorubicin) or 24 h (paclitaxel). Cells were harvested and luciferase activities were measured with the dual-reporter gene system (Promega, Mannheim, Germany) using a GLoMax Luminometer from Promega. For coexpression of pRF reporter plasmids and pCMV-GFP expression vectors, cells were simultaneously transfected with 0.3 μg of pRF and 0.1 μg of pCMV-GFP vectors for 24 h before the cells were lyzed for measurement of luciferase activities. Alternatively, luciferase activites from siRNA-transfected cells was measured 48 h after transfection of the siRNAs.

### Expression and purification of recombinant HuR proteins

Expression and purification of wild-type or different GST-HuR deletion mutants was performed as described previously [42].

### Preparation of cytoplasmic cell lysates

Preparation of cytosolic fractions from colon carcinoma cells was performed by using a protocol from Schreiber *et al*. [43].

### Biotin pull-down assay

10 μg of either *BamHI*-linearized plasmid pCR2.1-5ʹ-UTR-caspase-2 or *NotI*-linearized pDrive-hr-gapdh partial human reverse GAPDH (hrg) were *in vitro* transcribed using the “Riboprobe Combination System Kit” from Promega and biotin-labeled CTP (Invitrogen) following the manufacturer´s instructions. 20 μg of the biotinylated RNAs were conjugated to streptavidin-coupled agarose beads in incubation buffer (10 mM Tris-HCl pH 7.5, 150 mM KCl, 1.5 mM MgCl_2_, 0.5 mM DTT, 40 U/ml RNasin) at 4° C for 2 h under continuous rotation. Subsequently, 400 μg of cytoplasmic cell lysates were incubated with the beads under rotation for further 1 hour at 4° C. After several intensive washing steps with incubation buffer, the RNA-bound proteins were collected in 30 μl of Laemmli buffer and heated at 95° C for 5 min before analyzed by Western blot analysis with a HuR-specific antibody. Equal input material (20 μg) was confirmed by Western blotting using the same antibody and immunopositive signals were visualized by using ECL.

### Electrophoretic mobility shift assay (EMSA)

For monitoring binding affinity of endogenous HuR, a synthetic RNA oligonucleotide (IBA GmbH Göttingen) compassing a typical ARE from the 5ʹUTR of human cox-2 mRNA and referred to as COX-2-ARE-II was end labeled using T4 polynucleotide kinase and [γ^32^P] dATP (3 000 Ci/mmol) as described in a more detail by Akool *et al*. [44]. Binding of recombinant GST-tagged HuR proteins to full length 5ʹUTR-caspase-2, or to the F1 and F2 fragments of 5ʹUTR-caspase-2 was assessed by using the same *in vitro* transcribed 5ʹUTR caspase-2 probe used for biotin pull-down assay but instead of using a biotin-labeled RNA probe, the transcription was performed in the presence of [α^32^P] dUTP (3 000 Ci/mmol). The EMSA reaction was perfomed by incubation of 6 μg of cytoplasmic extract or, alternatively, the indicated amount of recombinant proteins and the radiolabeled RNA probe (30 000 cpm/reaction) for 30 min on ice in EMSA binding buffer (10 mM HEPES pH 7.6, 3 mM MgCl_2_, 40 mM KCl, 2 mM DTT, 5% glycerol and 0.5% Ipegal). Supershift analysis was performed by addition of 200 ng of antibody, or the same amount of mouse IgG, to the binding reaction for 30 min on ice prior to the addition of the radioactively labelled RNA probe. RNA-protein complexes were separated on 6% nondenaturing PAGE gels and run in Tris-borate-EDTA buffer.

### Measurement of cell death by FACS analysis

The quantification of the sub-G_1_ population of DLD-1 cells was performed with a FACSCanto II flow cytometer (Becton Dickinson, Heidelberg, Germany) as described previously [11]. Briefly, cells were seeded in 60 mm dishes and transfected with the relevant siRNAs as described previously [12]. 48 h after siRNA transfection, the cells were stimulated with vehicle or with different chemotherapeutic drugs for 24 h before collection by trypsinization. Z-VDVAD-FMK and Z-VAD-FMK were added 1 h before the chemotherapeutic drugs were administered. After removal of trypsin and washing of cells with PBS, cells were fixed overnight in absolute ethanol at −20° C. After centrifugation (300 × g for 2 min), cell pellets were resuspended in 0.3 ml hypotonic buffer containing 50 μg/ml propidium iodide (PI), 0.1% sodium citrate, 0.1% Triton X-100 and 10 μg/ml RNase A and incubated for 30 min at 37° C and directly analyzed thereafter by flow cytometry in linear mode using the FACSDiva Software (Becton Dickinson).

### Annexin-V/PI staining

The redistribution of plasma membrane phosphatidyl serine (PS) is a characteristic feature of apoptosis which was measured using the Rotitest-Annexin V kit (Carl Roth, Karlsruhe, Germany) following the instructions of the manufacturer. Briefly, cells grown on 60 mm dishes were collected by trypsinization and pooled with cell supernatants from corresponding culture dishes and 500 μl of this cell suspension was incubated with 2 μl of annexin-V-FITC stock solution for 15 min in the dark. After a short centrifugation (300 × g, 5 min), cells were washed in PBS before being resuspended in 0.5 ml of 1× binding buffer supplemented with either 1 μl PI (final: 1 μg/ml) or, alternatively, with 1 μl of Sytox Blue Dead Cell Stain (Invitrogen). Subsequently, Annexin V-FITC together with PI or Sytox Blue Dead Cell Stain was measured by flow cytometry and cell debris was excluded from the analysis.

### Confocal microscopy

Analysis of nucleo-cytoplasmic HuR distribution was performed by confocal microscopy as described previously [42]. Briefly, colorectal carcinoma cells were plated on microscope cover glasses in 12-well plates (neoLab Migge, Heidelberg, Germany) and stimulated with different chemotherapeutic drugs for individual time points. Afterwards, cells were fixed and permeabilized with 4% paraformaldehyde plus 0.25% Triton X-100 (AppliChem, Darmstadt, Germany) in PBS for 15 min. After blocking in 5% BSA in PBS, the staining was accomplished by incubation with a monoclonal anti-HuR antibody for 1 h at RT and after washing with PBS further incubated with Alexa Fluor 488 goat anti-mouse antibody. Nuclei were counterstained with 4ʹ,6-diamidino-2-phenylindole (DAPI) solution (Life Technologies) for 2 min and washed with PBS thereafter. Cover slips were mounted with Vectashield mounting medium (Alexis, Grünberg, Germany) and HuR was visualized using a LSM510 inverted laser scanning microscope (Carl Zeiss, Göttingen, Germany) and image acquisition was done using the ZEN2009 Light Edition software (Carl Zeiss).

### Statistical analysis

Data are given as means ± SD. For statistical analysis, the unpaired two-tailed *t*-test was applied (GraphPad Prism, GraphPad Software, Inc., La Jolla, CA, USA). *P* value ≤ 0.05 (^*^,^#^), ≤ 0.01 (^**^,^##^) and ≤ 0.005 (^***^, ^###^) were considered significant.

This work was supported by the Deutsche Forschungsgemeinschaft [EB 257/6-1, Excellence Cluster “Cardiopulmonary System (ECCPS)” EXC 147/1]. A.B. was financially supported by a scholarship from the DAAD and by the University of Khartoum (Sudan). U.N. was financially supported by a scholarship from the DAAD and by the Higher Education Commission of Pakistan (HEC).

## SUPPLEMENTARY MATERIALS FIGURES AND TABLES


