# Supplementary material for: Inhibition of IRES-dependent translation of caspase-2 by HuR confers chemotherapeutic drug resistance in colon carcinoma cells
Source: Oncotarget. 2018 Apr 6;9(26):18367–85. doi: 10.18632/oncotarget.24840 (PMC5915078; doi:10.18632/oncotarget.24840)
Supplement: Supplementary file 1 [file oncotarget-09-18367-s001.pdf]

Inhibition of IRES-dependent translation of caspase-2 by HuR confers chemotherapeutic drug resistance in colon carcinoma cells

SUPPLEMENTARY MATERIALS

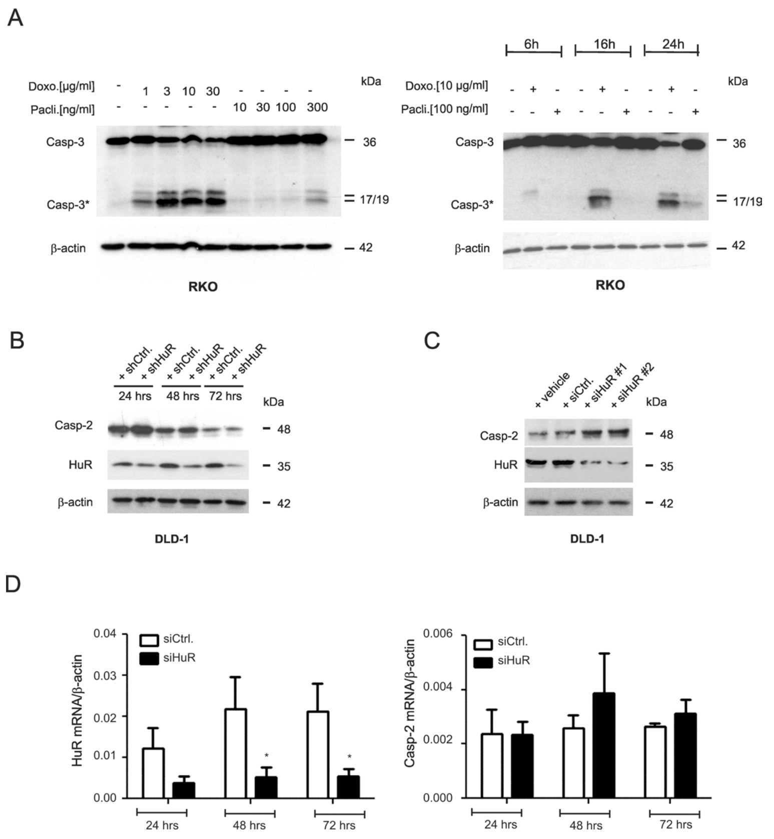

Supplementary Figure 1: (**A**) Concentration (left panel) and time-dependent (right panel) activation of caspase-3 cleavage by chemotherapeutic drugs. RKO cells were stimulated for the indicated time points with vehicle (−), or with the indicated doses of doxorubicin (Doxo.) or paclitaxel (Pacli.). Equal amounts of protein (20 µg) from total cell homogenates were subjected to SDS-PAGE and probed with anti-caspase-3 and anti-β-actin antibodies, respectively. The cleavage products of pro-caspase-3 (Casp-3) at 19 and 17 kDa are depicted by an asterisk (Casp-3*). Blots shown are representative for three independent experiments giving similar results. (**B**) Stable HuR knockdown by using Doxycycline- inducible HuR-specific shRNA expression vectors (shHuR) or, a non-targeting control shRNA expression plasmid. At the indicated time points after addition of doxycycline (1 µg/ml), cells were lyzed for total cell homogenates and subsequently, equal amounts of protein (20 µg) from DLD-1 transfectants were subjected to SDS-PAGE and probed with the indicated antibodies. β-actin was used as a loading control. Data shown are representative of two independent experiments giving similar results. (**C**) Transient HuR knockdown increases caspase-2 protein levels in DLD-1 cells. The increased levels of caspase-2 by HuR knockdown were assayed 48 h after siRNA transfection and validated with two different sets of siRNA duplexes complementary to distinct regions of the HuR mRNA (siHuR#1, siHuR#2) as described in Materials and Methods. β-actin was used as control for equal loading. (**D**) Time-course of HuR and caspase-2 mRNA levels after transient RNAi-mediated HuR knockdown. DLD-1 cells were transiently transfected with control duplexes (siCtrl.) or with siRNA duplexes of HuR (siHuR) for the indicated time periods before cells were harvested and extracted for total cellular RNAs. Changes in steady-state HuR mRNA (left panel) and caspase-2 mRNA levels (right panel) were measured by quantitative real-time PCR in a relationship to β-actin steady-state mRNA levels. Data represent means ± SD ($n = 3$), $^{*}P \leq 0.05$ siHuR. cells vs. siCtrl. cells.

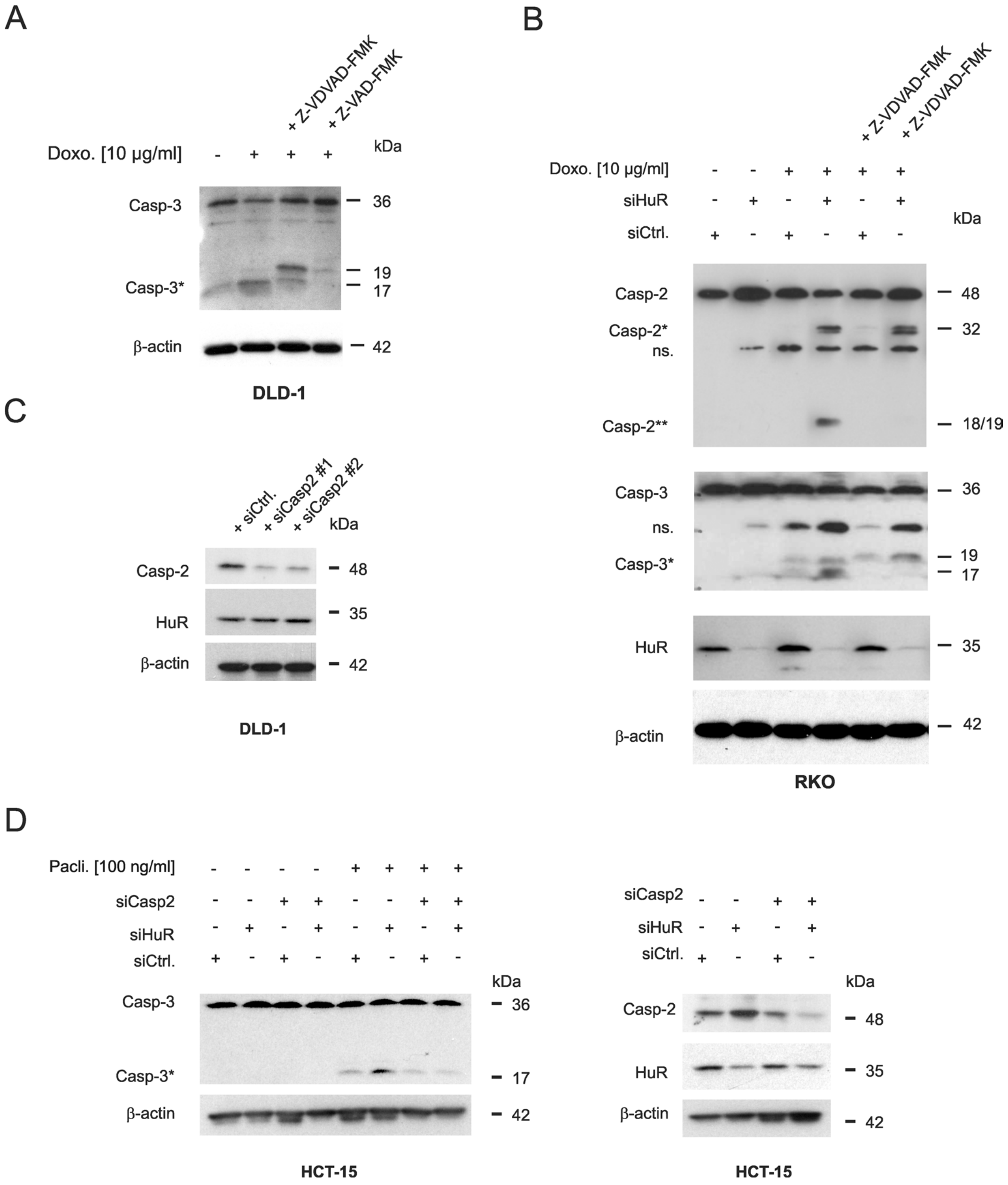

Supplementary Figure 2: Activation of caspase-3 cleavage by different chemotherapeutic drugs is affected by Z-VDVAD-FMK. (**A**) DLD-1 cells were stimulated with either vehicle (−), or with 10 µg/ml doxorubicin (+) either in the presence of Z-VDVAD-FMK or Z-VAD-FMK (both at 50 µM) which were added 1 h prior to doxorubicin. After 24 h, the cells were lyzed for total cell extracts and the processing of caspase-3 was monitored by Western blot analysis (cleavage products are indicated by asterisks) (**B**). Subconfluent RKO cells were transfected with control siRNA duplexes (siCtrl.) or with siRNA duplexes of HuR (siHuR) for 48 h before cells were stimulated with either vehicle (−), 10 µg/ml doxorubicin (Doxo.) either alone or in the presence of 50 µM of Z-VDVAD-FMK which was added 1 h prior to the administration of doxorubicin. After 24 h cells were lyzed for total cell extracts and the processing of caspase-2 and caspase-3 (cleavage products of caspases are indicated by asterisks and by double astersiks) and the knockdown efficiency of HuR was subsequently confirmed by Western blot analysis. The positions of nonspecific bands (ns.) are indicated. Representative data of three independent experiments are shown. (**C**). Knockdown of caspase-2 by two different siRNAs (siCasp2#1, siCasp2#2), complementary to distinct regions of the coding region of caspase-2 mRNA was assayed 48 h after transfection. HuR was used as a negative control and β-actin as a loading control. Blots shown are representative for two independent experiments giving similar results. (**D**) HCT-15 cells were transfected with control siRNA duplexes (siCtrl.) or with siRNA duplexes of caspase-2 (siCasp2) or HuR (siHuR) or alternatively, double transfected with HuR plus caspase-2-specific siRNA duplexes for 48 h before cells were stimulated with paclitaxel as indicated. 48 h after stimulation the processing of caspase 3 was determined by Western blot analysis (cleavage products of caspase-3 are indicated by an asterisk). β-actin was used as loading control.

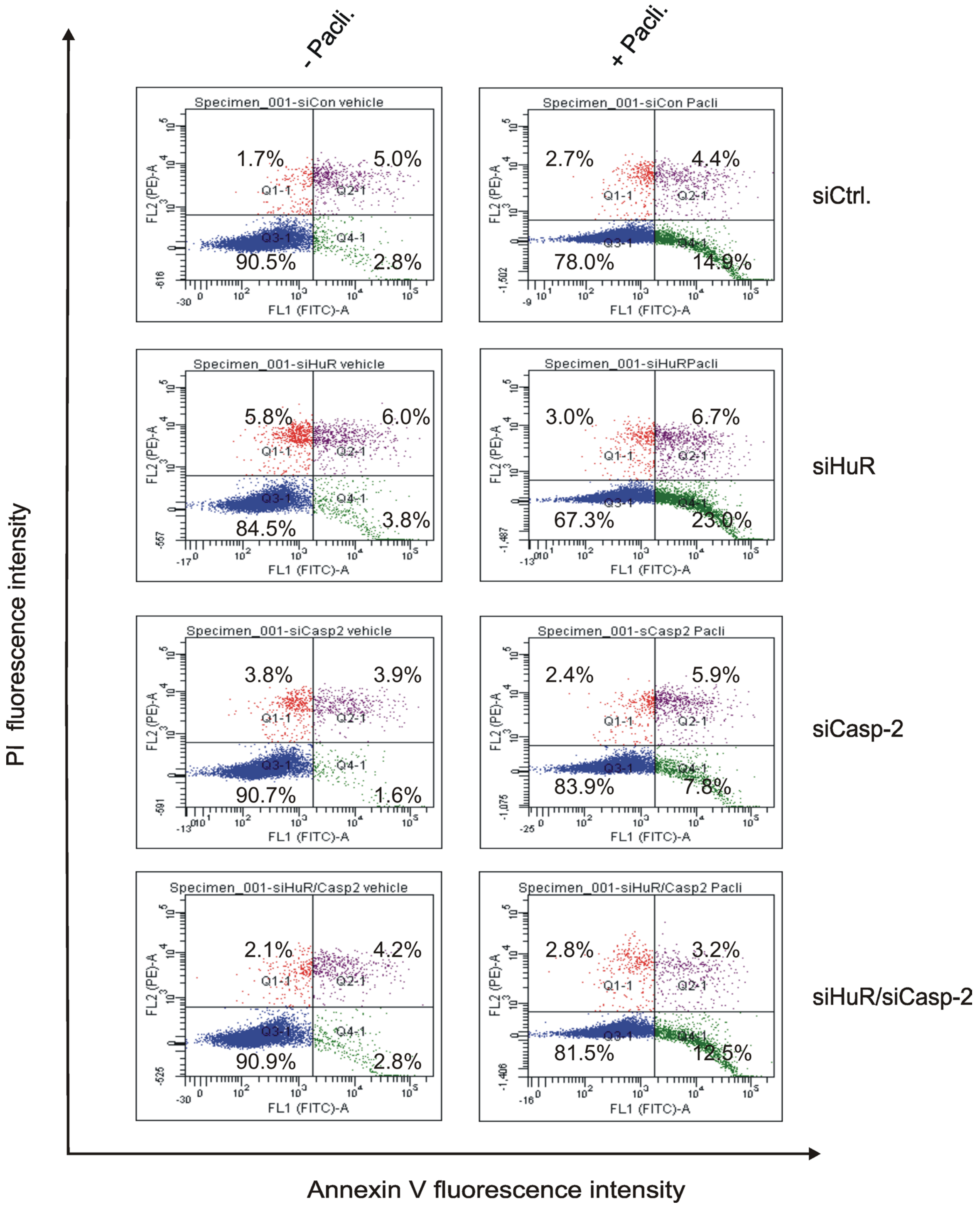

Supplementary Figure 3: DLD-1 cells were transfected with control siRNA duplexes (siCtrl.) or with siRNA duplexes targeting caspase-2 (siCasp2) or HuR (siHuR) or alternatively, double transfected with HuR plus caspase-2-specific siRNA duplexes (siHuR/siCasp2) for 48 h before cells were treated in the absence (− Pacli.), or presence of 100 ng/ml paclitaxel (+ Pacli.). After further 24 h, cells were subjected to flow cytometric analysis for determination of different cell death parameters by using annexin-V-FITC in combination with PI staining as described in the Materials and Methods section. Representative data of three independent experiments are shown.

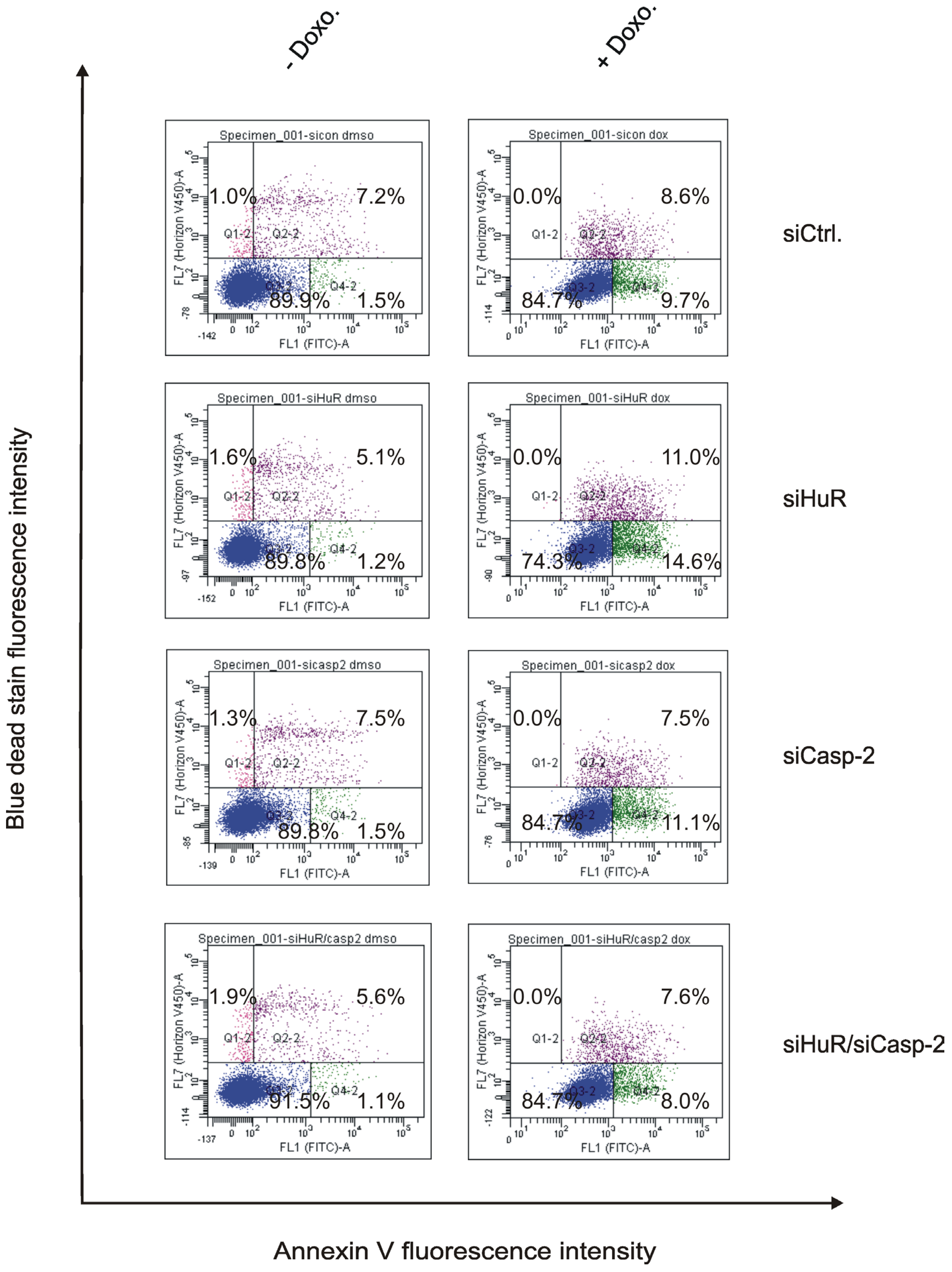

Supplementary Figure 4: DLD-1 cells were transfected with control siRNA duplexes (siCtrl.) or with siRNA duplexes targeting caspase-2 (siCasp2) or HuR (siHuR) or alternatively, double transfected with HuR plus caspase-2-specific siRNA duplexes (siHuR/Casp2) for 48 h before cells were treated in the absence (− Doxo.), or presence of 10 µg/ml doxorubicin (+ Doxo.). After further 24 h, cells were subjected to flow cytometric analysis for determination of different cell death parameters by using annexin-V-FITC in combination with blue dead staining as described in the Materials and Methods section. Representative data of three independent experiments are shown.

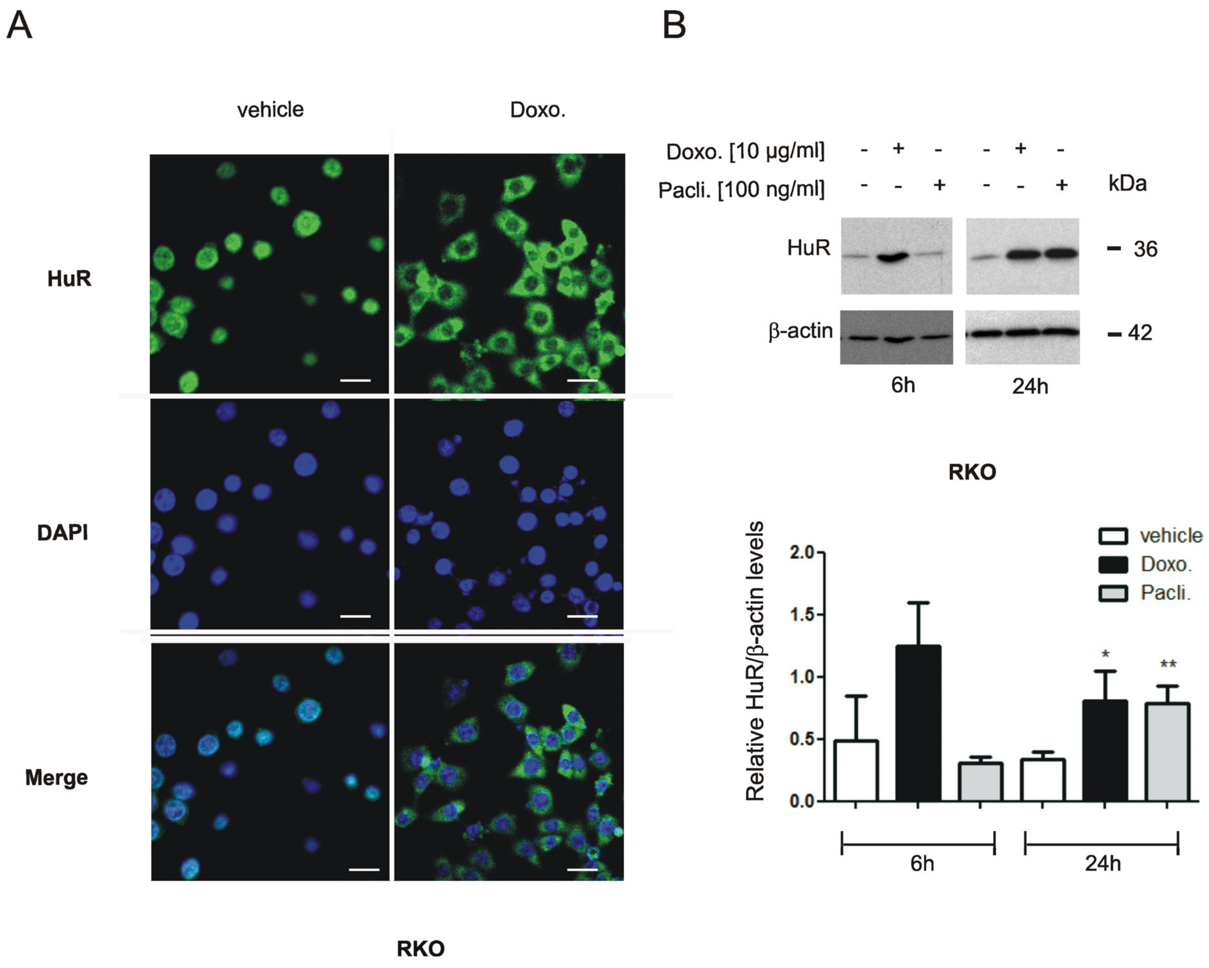

Supplementary Figure 5: Modulation of nucleo-cytoplasmic HuR shuttling by chemotherapeutic drugs. (**A**) Serum-starved RKO cells were treated for 6 h with either vehicle, or with 10 µg/ml doxorubicin (Doxo.) before cells were fixed and successively stained with anti-HuR and anti-mouse Alexa-488 antibodies. Thereafter, DAPI was added to counterstain cell nuclei (blue panel), bar: 50 µm (**B**). Time-dependent increase in cytoplasmic HuR content by chemotherapeutic drugs. Serum-starved RKO cells were treated with doxorubicin (Doxo.) or, paclitaxel (Pacli.) as indicated, before cells were lyzed for cytoplasmic cell lysates and cytoplasmic HuR levels were subsequently monitored by Western blot analysis. Loading of equal amounts of cytoplasmic extracts was ascertained by reprobing the blots with anti-β-actin antibody. Values at the bottom represent means ± SD ($n = 3$) $^{*}P \leq 0.05$, $^{**}P \leq 0.01$ compared with the corresponding vehicles.

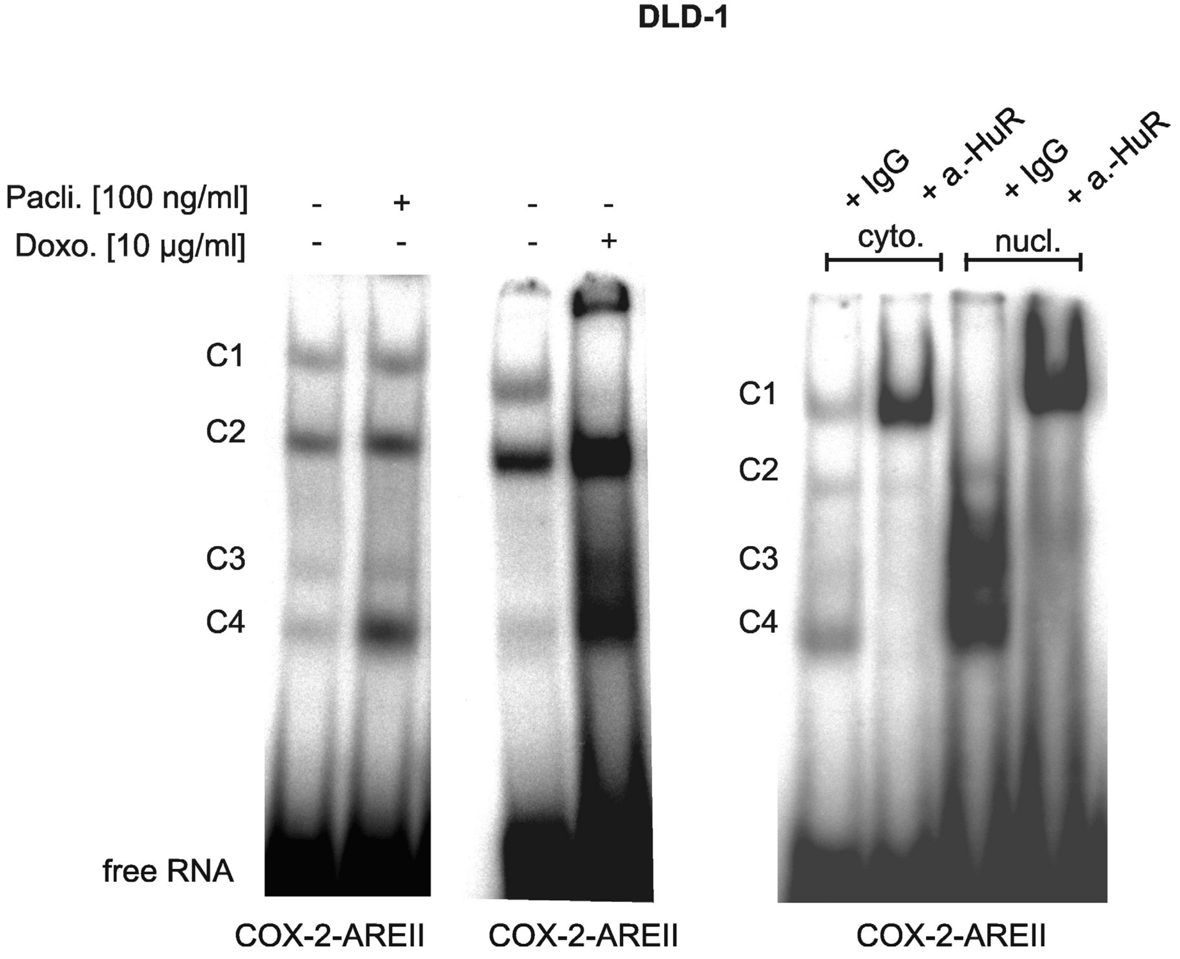

Supplementary Figure 6: Chemotherapeutic drugs increase the constitutive RNA binding of HuR-containing complexes to an AU/U-rich element (ARE) from the 3´UTR of human COX-2 mRNA. Cytoplasmic extracts (5 µg) from DLD-1 cells treated either with vehicle (−), for 6 h with doxorubicin (Doxo.) or, for 24 h with paclitaxel (Pacli.) at the indicated doses were added to the EMSA reaction together with the ^{32}P-labeled RNA probe encompassing an ARE (ARE-II) from the human COX-2-3′UTR. RNA-protein complexes (C1–C4) were resolved from unbound RNA by nondenaturing PAGE. The EMSA shown is representative for three independent experiments giving similar results. *(right panel).* Supershift analysis implicates that HuR is a main constitutent of cytoplasmic (cyto.) and nuclear (nucl.) complexes C2, C3 and C4, respectively.

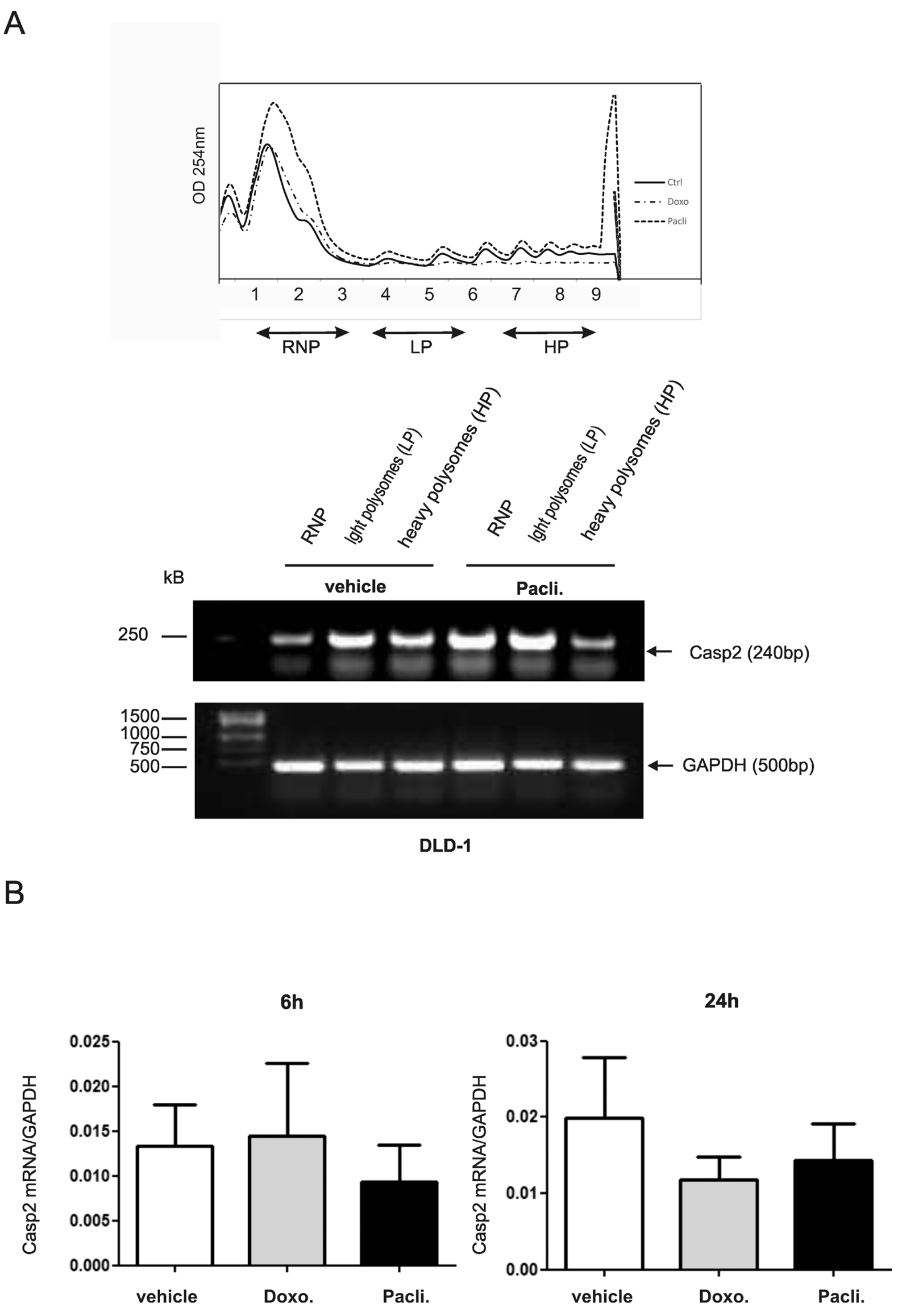

Supplementary Figure 7: (**A**) Paclitaxel causes a shift from heavy polysomes to the translational inactive sub-polysomal fraction containing ribonucleoproteins (RNP). DLD-1 cells were treated for 24 h with 100 ng/ml paclitaxel (Pacli.) or 10 µg/ml doxorubicin (Doxo.) and lyzed for total cell homogenates. Subsequently, the cell lysates were subjected to polysomal fractionation. RNA from pooled fractions from either RNP (1–3), light polysomes (LP, 4–6) or heavy polysomes (HP, 7–9) was isolated and the content of caspase-2 and GAPDH mRNA determined by semiquantitative reverse transcription (RT)-PCR by using gene-specific primers. The length of corresponding PCR products was determined by a DNA ladder. Data shown are representative of two independent experiments giving similar results. (**B**) DLD-1 cells were treated for 6 h (left panel) or 24 h (right panel) with either vehicle, or with doxorubicin (Doxo.), or with paclitaxel (Pacli.) before cells were harvested and extracted for total cellular RNAs. Changes in caspase-2 mRNA levels were determined by quantitative RT-PCR using GAPDH mRNA for normalization. The values represent means ± SD ($n = 3$).

A.

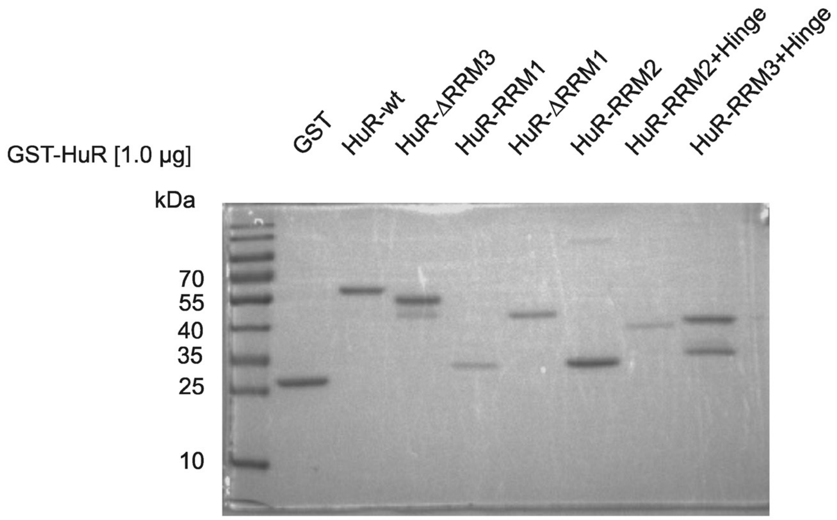

B

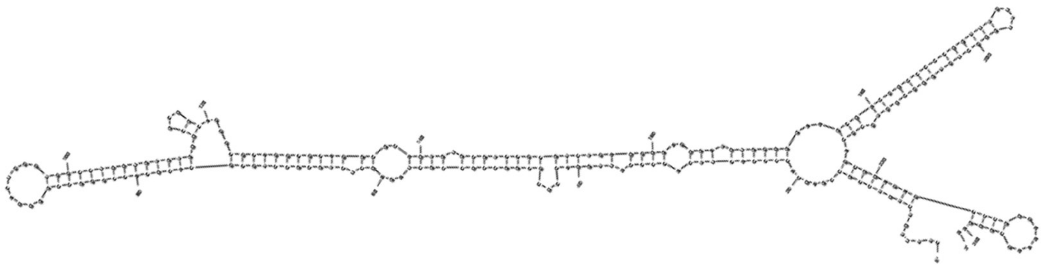

Supplementary Figure 8: (**A**) Loading control for expression of ectopically expressed GST-HuR constructs. In each lane 1 µg of GST or GST-HuR fusion protein (schematically summarized in Figure 8C) was loaded on SDS-PAGE and proteins were subsequently visualized by Coomassie-blue staining. (**B**) Predicted structure of the human casp2-5′UTR (NM_032982) using *mfold*.
